# Novel Tl(III) complexes containing pyridine-2,6-dicarboxylate derivatives with selective anticancer activity through inducing mitochondria-mediated apoptosis in A375 cells

**DOI:** 10.1038/s41598-021-95278-y

**Published:** 2021-08-03

**Authors:** Sara Abdolmaleki, Mohammad Ghadermazi, Alireza Aliabadi

**Affiliations:** 1 Department of Chemistry, Faculty of Science, University of Kurdistan, Sanandaj, Iran; 2 Pharmaceutical Sciences Research Center, Health Institute, School of Pharmacy, Kermanshah University of Medical Sciences, Kermanshah, Iran

**Keywords:** Biochemistry, Cancer, Chemistry

## Abstract

Three novel Tl(III) complexes (C1), (C2) and (C3) were synthesized using the one-pot reactions of pyridine dicarboxylic acid derivatives, 2-aminobenzimidazole and/or 4-aminopyridine, and also thallium(III) nitrate trihydrate metal salt. The structure of all three complexes was determined by the single-crystal X-ray diffraction. C1 and C2 were realized to be isostructural with disordered square anti-prismatic geometry and for C3 arrangement of the distorted tricapped triangular prism was proposed. Cyclic voltammetry measurements on the complexes exhibited that formal potential values are more positive for C1 (E^0^ˊ 0.109 V) and C3 (E^0^ˊ 0.244 V) compared to C2 (E^0^ˊ –0.051 V), versus Ag/AgCl under argon. Moreover, cytotoxicity of the compounds was evaluated in vitro against two cancer cell lines including a human melanoma (A375), a human colon adenocarcinoma (HT29), and also one normal cell human foreskin fibroblast (HFF). The selective and potent cytotoxicity effect was exhibited by C1 and C3 on cancer cell lines. The apoptosis through a caspase-dependent mitochondrion pathway was confirmed by ROS production, MMP reduction, p53 activation, Bax up-regulation, and Bcl-2 down-regulation, cytochrome *c* release, procaspase-9, and 3 expression, for A375 cells treated to C1 and C3. According to similar cellular uptake of the complexes in A375 cell line, the generation of ROS was considered as an effective agent to justify the inhibition effect C1 and C3 on mentioned cells. Furthermore, arresting the cell cycle in the G2-M phase and inducing apoptosis were indicated by these two complexes.

## Introduction

The unique chemical and biological properties of main-group elements have put them in the spotlight^1^. The studies have shown that many metal ions from these groups can be effective in the hydrolysis of peptides and proteins and even some proteolytic enzymes require such ions for their activities^2^. Among them, metal ions of group 13 have gained a special position due to potential applications in the treatment and diagnosis of diseases^3^. Thallium is the latest metal introduced in this group and because of high toxicity, poor investigations have been performed on it^4^. Thallium can be found in the environment in two oxidation states of Tl(I) and Tl(III) though the main form is Tl(I). Thallium in this oxidation state represents a high similarity to potassium cation^2,5^. The toxicity of Tl(I) is related to its interference in the vital potassium-dependent processes so that it can substitute potassium in the (Na^+^-K^+^)-ATPase and represent a high affinity for sulfhydryl groups from proteins and other biomolecules.

From the point of medicine, thallium has been reported effective in the treatment of diseases such as syphilis, tuberculosis, and malaria. Such effectiveness is related to the high toxicity of this metal. On the other hand, the isotope^20^^1^Tl has been applied in imaging of the myocardium and tumors^6,7^ and indicated achievements for recognizing the proliferation of small lung cancer^4^.

The chemical properties of Tl(I) such as the presence of lone electron pair, large size, salts that are often anhydrous, diverse coordination numbers, and geometries have caused that, despite high toxicity, its coordination compounds be especially regarded^8^. The evaluations on the toxicity rate of thallium show that coordinating with some organic molecules has a significant role in the reduction of its toxicity rate^9^. So that some of the thallium(III) porphyrins can represent favorable medical applications^1^. Accordingly, M. Mimouni et al., in 2014^10^ designed thallium complexes from monensin and lasalocid and evaluated their antibacterial, antifungal, and antitubercular activities. In this study, favorable pharmaceutical applications without a high risk of toxicity were recognized for the complexes^11^. Also, antimicrobial applications have been proven in some thallium compounds such as Tl acetate and Tl carbonate^6^. In another study, the anti-tumor activity of *para*-substituted tetraphenyl porphyrin Tl(III) salicylate complexes was evaluated and a strong inhibition effect (up to 90%) of 5-SSATl(III)t(4-OCH3)PP was exhibited against all four human cancer cell lines tested including MCF-7, THP-1, PC-3 and A549^1^. In 2013, A. S. El-Tabl et al., synthesized Tl(I) complexes from 2-(2-(4-carboxyphenyl)guanidino)acetic acid as ligand and exhibited a potent anti-proliferative effect on the MCF-7 cell line compared to the standard drug^12^. Accordingly, new hopes emerged for the continuation of biological studies on thallium compounds^13^. But, besides medicine fields, thallium and its compounds have indicated applications as semiconductors, catalysts, dyes, and pigments^14^. Such diverse uses led to consider thallium compounds in various areas of knowledge^15^.

Some studies exhibited that organic ligands can reduce Tl(III) to Tl(I) ^SPS:refid::bib1616^. It is a very important topic in biochemistry because the oxidation state of the metal has a significant role in the biological properties of inorganic compounds^17^. For example, thallium(III) has a higher toxicity than Tl(I)^18^. But besides the role of metal and its oxidation state, the nature of ligand coordination should not be ignored to evaluate the cytotoxicity effects of metallic complexes. It has been proven that versatile anionic ligands including carboxylate groups with diversity in the coordination modes are usually favorable options to design metal complexes and an assay of their medicine activity^8^. In 2005, M. Rafizadeh et al., synthesized the thallium(I) complex containing 2,6-Pyridinedicarboxylic acid (pydcH_2_) and reported its crystal structure^19^. Investigations have indicated that among various diacids, pyridine-2,6-dicarboxylic acid^20^ has the most favorable biochemical and chemical features^21^.

Therefore in the present study, three novel thallium complexes were designed from pyridine dicarboxylic acid derivatives and were characterized by single-crystal X-ray diffraction. In the next step, the electrochemical and anticancer properties of the compounds and as well as the correlation between these two activities were evaluated. Then, detailed studies were performed to recognize the apoptosis pathway.

## Results

### X-ray diffraction studies of the compounds

**Figure 1:**
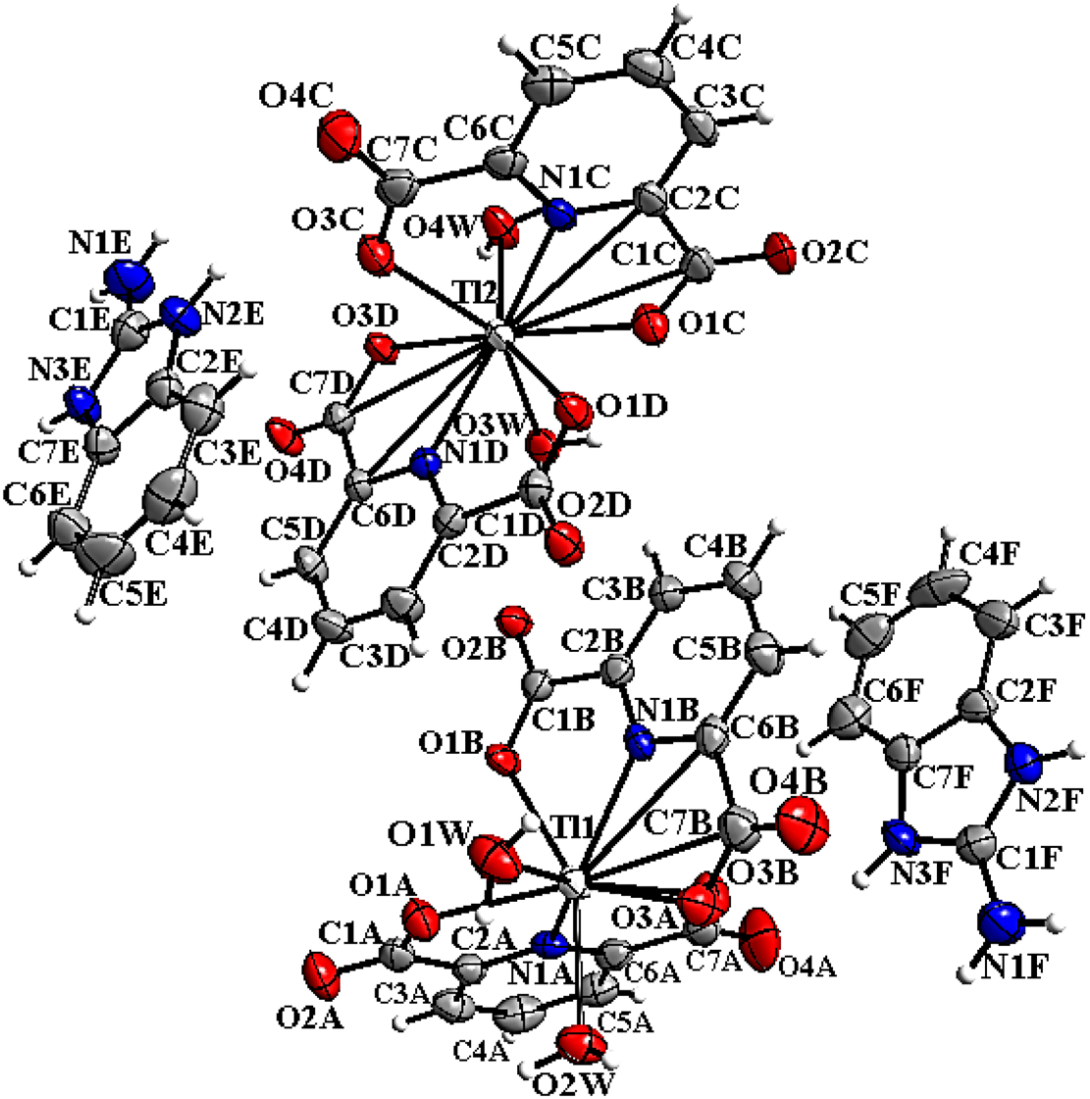
Molecular structure of C1 showing the atom numbering scheme with 50% probability of thermal ellipsoids.

**Figure 2:**
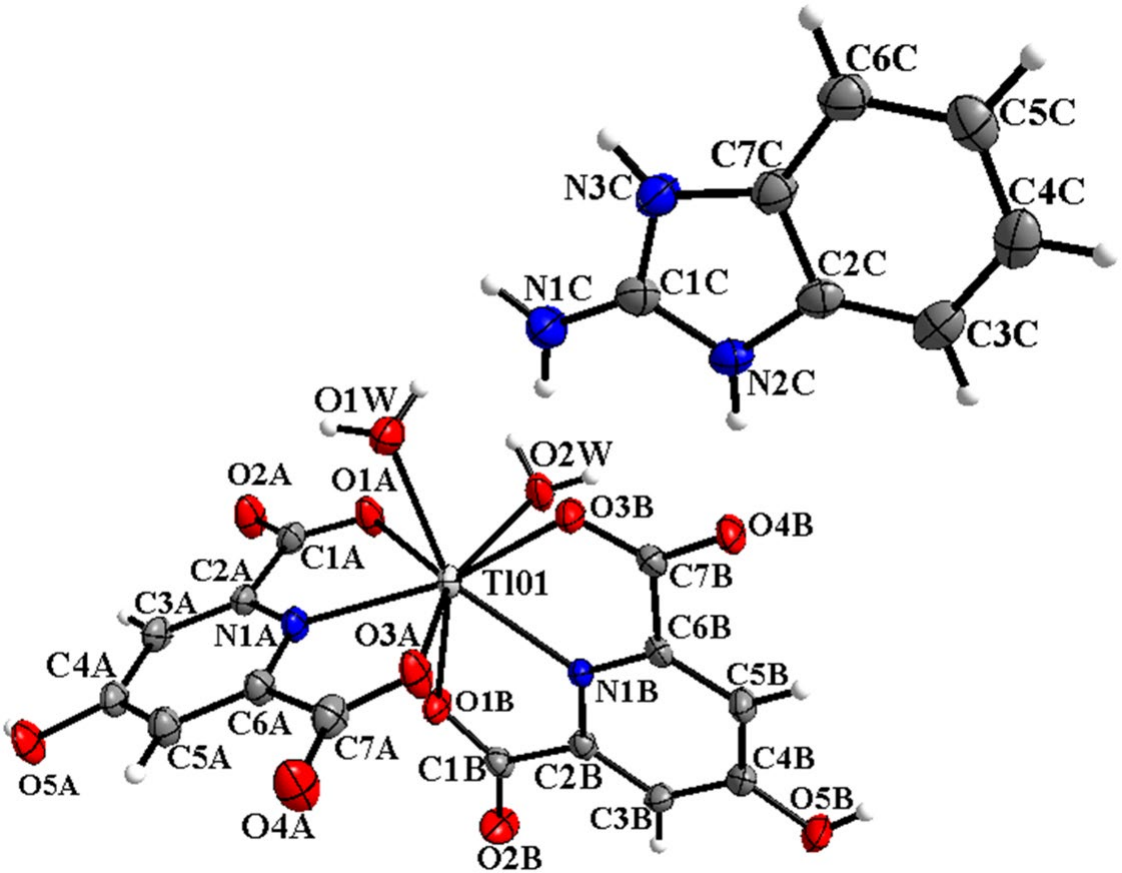
Molecular structure of C2 showing the atom numbering scheme with 50% probability of thermal ellipsoids.

The synthesis route of complexes is shown in Scheme S1. The crystallographic data of the complexes are represented in Table 1. The selected bond lengths, angles, and intermolecular hydrogen bond parameters are shown in Tables S1 and S2, respectively. Both compounds C1 and C2 were crystallized in the triclinic crystal system with the *P-1* space group. These two complexes were isostructural and contain anionic units [Tl(pydc)_2_(H_2_O)_2_]^−^ for C1 and [Tl(hypydc)_2_(H_2_O)_2_]^−^ for C2. Also, extranuclear cations (2-abH)^+^ and water molecules co-crystallized are observed along with complex units in these structures. In the anionic parts of both C1 and C2 respectively, (pydc^2−^) and (hpydc^2−^) molecules coordinated to one Tl(III) atom, through two oxygen and one nitrogen atoms, and two water molecules were also available in the coordination sphere (Figs. 1 and 2). Therefore, disordered square anti-prismatic geometry was indicated for Tl(III) eight coordinated in both complexes. In 2004 M. Ranjbar et al., reported a Tl(III) complex from pyridine-2,6-dicarboxylic acid that was seven coordinated, and the distorted pentagonal bipyramidal arrangement was observed for it^22^. This structure was completely different from C1 and C2.

**Table 1:** Crystallographic and structural refinement data for the complexes.

	C1	C2	C3
Empirical formula	C42H50N10O27Tl2	C21H30N5O18Tl	C26H28N5O17Tl
Formula weight	1535.66	844.87	886.90
Crystal system	Triclinic	Triclinic	Monoclinic
Space group	P-1	P-1	P21/c
a/Å	10.6725 (2)	9.7786 (4)	9.9096 (1)
b/Å	16.1679 (5)	10.4739 (3)	19.3465 (2)
c/Å	16.6143 (4)	14.6356 (3)	16.1107 (2)
α (°)	106.316 (3)	95.574 (2)	90
β (°)	103.263 (2)	104.856 (3)	91.855 (1)
γ (°)	93.232 (2)	98.870 (3)	90
Volume (Å^3)^	2655.49 (12)	1416.81 (8)	3087.06 (6)
Z	2	2	4
Crystal size (mm)	0.36 × 0.22 × 0.07	0.29 × 0.21 × 0.05	0.23 × 0.17 × 0.14
Shape	Prisms	Prisms	Prisms
Color	Yellow	Colorless	Colorless
Density (calc.) (g cm^−1^)	1.921	1.980	1.908
Absorption coefficient (mm^–1^)	6.160	5.79	5.32
F(000)	1500	832	1744
θ range for data collection (°)	3.238 to 24.711	3.697 to –32.598°	3.230 to 26.372
Index range	–12 ≤ h ≤ 12 − 16 ≤ k ≤ 19 − 19 ≤ l ≤ 19	–13 ≤ h ≤ 14 − 14 ≤ k ≤ 15 − 22 ≤ l ≤ 19	–12 ≤ h ≤ 12 − 21 ≤ k ≤ 24 − 19 ≤ l ≤ 20
Reflections collected	17,380	17,087	46,810
Independent reflections	8147 [R(int) = 0.028]	9420[R(int) = 0.030]	6297 [R(int) = 0.035]
Data/restraints/parameters	12,372 / 0 / 753	9360 / 0 /429	26,043 / 0 / 431
Final R indices [I > 2sigma(I)] R_1_, wR_2_	0.023, 0.059	0.029, 0.058	0.020, 0.063
R indices (all data) R_1_, wR_2_	0.026, 0.057	0.035, 0.055	0.023, 0.06
Goodness of fit on F^2^(S)	1.03	1.01	0.96
Largest diff peak and hole (e ·Å^–3^)	0.92, − 0.52	1.02, − 0.81	1.00, − 0.67

Hydrogen bonds containing O–H···O, N–H···O and C–H···O with D···A distances ranging from 2.63(14) to 3.49(15) Å for C1 (Figure S2a) and 2.64(14) to 3.39(12) Å for C2 (Figure S3a) lead to the formation the supramolecular networks in these compounds^23^. These bonds were created between carboxylate moieties, water molecules, and cations (2-abH)^+^. The strongest hydrogen bonds seem to be N(2)–H(2)···O(11) [*x − 1, y, z*] and O(5 W)–H(5WB)···O(8 W) [*− x* + *1, − y* + *1, − z*] for C1; and O(5B)–H(5B)···O(1 W) and O(12 W)–H(12A)···O(2D) [*x* + *1, y, z* + *1*] for C2. These interactions lead to the appearance of cyclic hydrogen bonding synthons as *R* $$\begin{array}{c}4\\ 6\end{array}$$(16), *R* $$\begin{array}{c}3\\ 3\end{array}$$(10) and *R* $$\begin{array}{c}3\\ 4\end{array}$$(8) for C1 (Figure S2b); and *R* $$\begin{array}{c}2\\ 3\end{array}$$(8), *R* $$\begin{array}{c}2\\ 2\end{array}$$(8) and *R* $$\begin{array}{c}4\\ 4\end{array}$$(8) for C2 (Figure S3b)^24^.

Besides hydrogens bonding, stacking interactions containing π-π, C–O···π and C–H···π are also shown in these structures which help to form more connections between anionic and cationic moieties and subsequently grow the crystal. C–O···π interactions occur for C1 between C(1D)–O(2D) and C(1B)–O(2B) respectively, with (N1B, C2B–C6B), and (N1D, C2D–C6D) rings at distances 3.26(11) and 3.13(12) Å, and angles 91.6(2) and 93.7(3)°, and also, C(7D)–O(4D) with (2-abH)^+^ at distance 3.18(11) Å, and angle 87.8(2)° (Figure S4). These interactions were observed for C2 between N(2C)–H(2C) with (N1B, C2B-C6B ) and C(1B)–O(2B) with (2-abH)^+^ at distances 3.28(11) and 3.50(11) Å, and angles 82.8(2) and 84.5(3) °, respectively (Figure S5).

**Figure 3:**
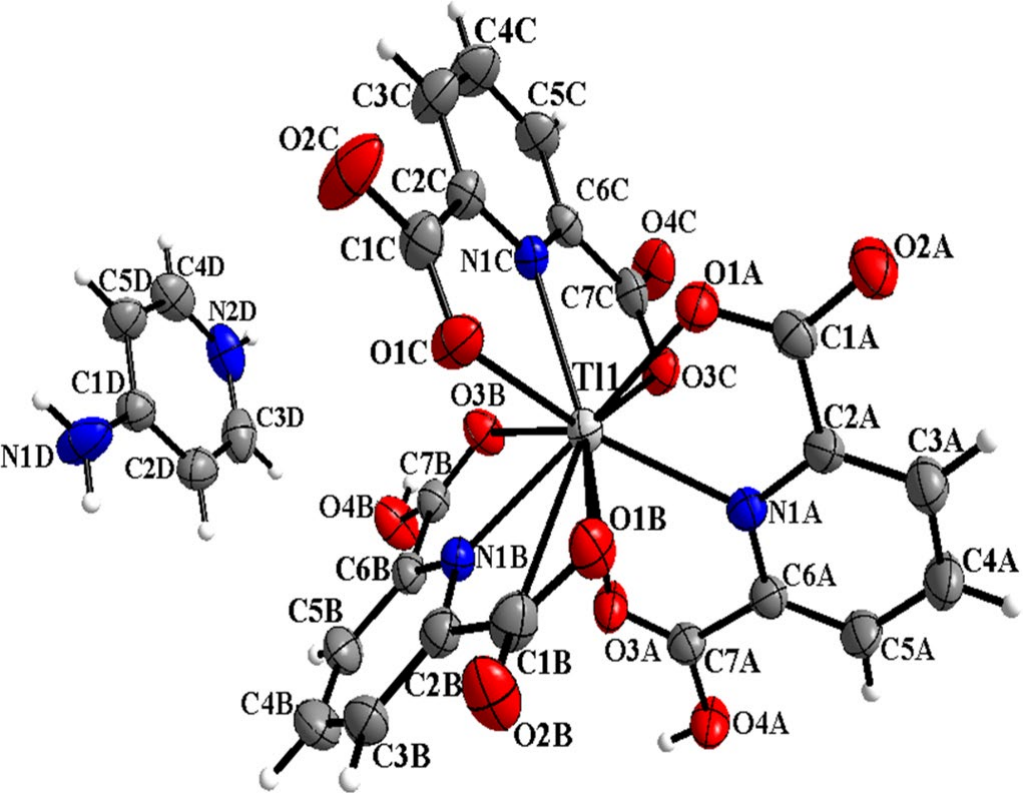
Molecular structure of C3 showing the atom numbering scheme with 50% probability of thermal ellipsoids.

Compound C3 and the coordination polyhedron around Tl(III) atom is shown in Fig. 3. This complex was crystallized in a monoclinic *P2*_*1*_*/c* space group with four moieties in the unit cell. In the structure C3, one anionic unit [Tl(pydc)(pydcH)_2_]^−^, a (4-apyH)^+^ cationic moiety, and five water molecules uncoordinated were observed. In this compound, three pyridine-2,6-dicarboxylate molecules coordinate to Tl(III) in tridentate mode through two oxygen atoms and one nitrogen atom so a prism with three caps of three nitrogen atoms on its faces is exhibited and distorted tricapped triangular prism geometry is suggested for Tl(III) nine coordinated. According to the best of our knowledge and data obtained from the Cambridge crystallographic data centre, nine coordination number has not been observed for Tl(III) complexes synthesized of pyridine dicarboxylic acid derivatives until now^25,26^ and it is reported for the first time by us. In this structure, the difference of bond length C7A–O3A = 1.20(13) and C7B–O3B = 1.20(13) with C7A–O4A = 1.30 (13) and C7B–O4B = 1.30(13) confirmed the presence of COOH groups.

The sum of bond angles, N1B–Tl1–N1C = 109.9 (7), N1A–Tl1–N1B = 116.6 (7) and N1A–Tl1–N1C = 130.5 (7) ° is exactly equal to 357.1° which proves that N1A, N1B, and N1C atoms form a planar triangle around Tl(III). Also, two other triangles are observed from three atoms containing O1A, O1B, and O1C, and three atoms containing O3A, O3B, and O3C, on two sides of the first triangle (Figure S6). The angles between oxygens indicate that they are eclipsed. The intra- and intermolecular hydrogen bonds include N–H···O and C–H···O with D···A distances ranging from 2.72(13) to 3.52(14) Å for this structure. The strongest hydrogen bonds can be N(4D)–H(4D)···O(2C) [*x* + *1, y, z*], O(4 W)–H(4WB)···O(2A) and O(4 W)–H(4WA)···O(4C) [*− x* + *1, − y* + *1, − z*] for C3. The presence of water molecules in this structure increases the probability of the formation of hydrogen bonds and also has a significant role to create water clusters, three-dimensional networks, and following the stability of the crystal lattice. The views of (H_2_O)_n_ tetrameric and pentameric water clusters are shown for C3 in Figures S7 and S8^27^.

### Electrochemical properties

**Figure 4:**
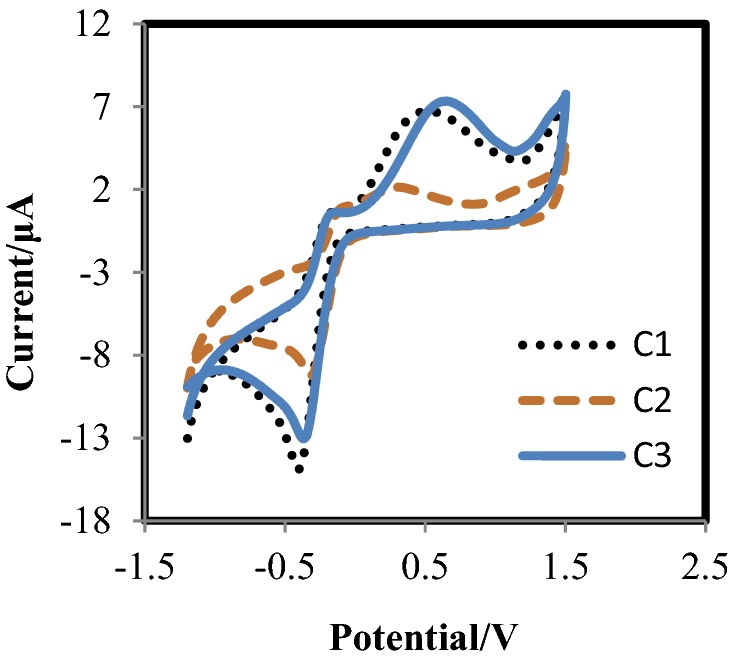
Cyclic voltammogram of complexes c = 4 × 10^–3^ mol/dm^3^ in H_2_O medium, KCl (0.1 M) at GCE, scan rate 50 V/s.

The electrochemical behavior of the complexes and also their ligands was studied by the cyclic voltammograms in the potential range of + 1.5 to –1.5 V and a scan rate of 50 Vs^–1^. The voltammograms are exhibited in Fig. 4. Redox couples at E^0ˊ^ 0.109, –0.051, and 0.244 V were obtained for C1, C2, and C3, respectively. The electrochemical oxidation of all three complexes seems be a one-stage process which includes irreversible couples with E_C_ = –0.363 V, E_A_ = 0.472 V for C1, E_C_ = –0.271 V, E_A_ = 0.220 V for C2 and E_C_ = –0.337 V, E_A_ = 0.581 V for C3. The peaks can be interpreted according to the Tl(III)/Tl(I) redox system for complexes^9^. As can be seen, the oxidation potential of C1 and C3 is at more positive values compared to C2. The investigations indicated that ligands do not have remarkable electrochemical reactivity. Therefore, redox couples are related to only the metal centers in complexes^27,28^.

### Cytotoxic property

Before the study on cytotoxicity of the complexes, their stability was confirmed by UV–Vis spectral analysis at different times in a solution containing DMSO/H_2_O (Figure S9). Then the inhibitory effect of the compounds was evaluated by MTT assay against A375, HT29, and HFF cell lines (Figure S10, Table 2). The anti-proliferative effect of ligands was indicated weaker than oxaliplatin on all three cell lines similar to our previous papers^29^. Many studies have proven that inorganic compounds were usually exhibited a more potent cytotoxic effect compared to their organic ligands on cancer cells. It seems that sometimes coordination can be effective in the improvement of anticancer properties of synthetic chemical compounds^30,31^.

**Table 2:** Cytotoxicity (IC_50_ ± SEM, μM) of the compounds against A375, HT29 and HFF cell lines for 48 h.

	A375	HT29	HFF
C1	81.45 ± 1.02	354.36 ± 0.71	> 500
C2	421.13 ± 0.55	486.25 ± 0.69	> 500
C3	7.23 ± 0.93	193.18 ± 1.12	> 500
pydcH2	442.64 ± 1.22	430.23 ± 2.09	> 500
hpydcH2	> 500	> 500	> 500
2-ab	> 500	> 500	> 500
4-apy	491.87 ± 1.56	> 500	> 500
Oxaliplatin	331.03 ± 2.69	323.13 ± 2.87	429.23 ± 2.32

No significant effect was observed on cells tested for metal salt.

In this study, A375 cells exhibited higher sensitivity to C1 (IC_50_ = 81.45 μM, Viability inhibition = 55.44%) compared to oxaliplatin (IC_50_ = 331.03 μM, Viability inhibition = 46.79%) while cytotoxic effect for C2 (IC_50_ = 421.13 μM, Viability inhibition = 35.28%) was indicated weaker than C1 and oxaliplatin on these cells. Here, the results confirmed that C3 (IC_50_ = 7.23 μM, Viability inhibition = 69.62%) has the most significant effect of inhibition compared to other compounds on A375 cells. Also, the most cytotoxicity on HT29 cell line is exhibited by C3 (IC_50_ = 193.18 μM, Viability inhibition = 53.12%). On the other hand, all three complexes indicated the weak cytotoxicity toward human foreskin fibroblast cells (IC_50_ > 500 μM, Viability inhibition = 20.72–31.18%). The potent inhibitory effect on A375 cells merits C1 and C3 for detailed evaluations. According to the best of our knowledge, reports on the anticancer effect of thallium complexes are rare and there are limited studies in this field. In this study, since thallium is located in the center of all three complexes, and also ligands coordinated to thallium are pyridine dicarboxylic acid derivatives, the obtained results can highlight the structure − activity relationship. Paying attention to these points provides useful information for designing more potent and selective pharmaceutical compounds with fewer side effects in the field of cancer treatment.

### Cellular uptake of Tl

The cellular uptake of pharmaceutical compounds has usually an important role in the rate of their biological activity^32,33^. Here, Tl content was determined in A375 cells treated with Tl(NO_3_)_3_.3H_2_O and C1–C3 to investigate whether the type of ligand and coordination geometry can be effective on cellular uptake. A375 cells treated with all three complexes for 24 h exhibited a remarkable increase in the cellular concentration of thallium compared with Tl(NO_3_)_3_—treated cells. The results of this evaluation clearly showed that weak cytotoxicity C2 compared to C1 and C3 is related to its inherent properties since all complexes are drawn easily into the cells (Figure S11). When we evaluated cellular uptake of C3 in HFF cells (Figure S12), it was realized that the cellular concentration of thallium was less in normal cells than cancer cells (A375), but the amount of thallium in these cells was not zero; therefore it cannot be concluded with certainty that the reason for the low efficacy of the C3 against normal cells is only due to its low penetration into these cells.

These results directed our study toward the determination of ROS in A375 and HFF cells following treatment with the complexes.

### Intracellular ROS measurement

**Figure 5:**
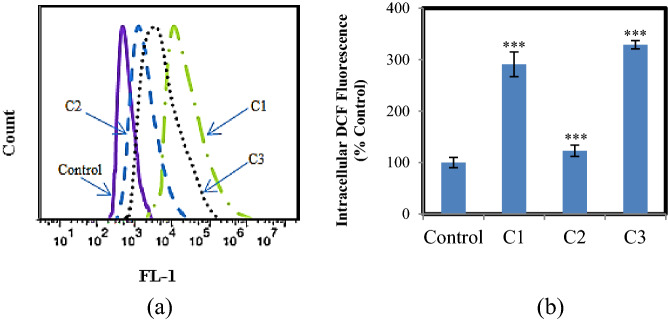
(**a**) Intracellular ROS was detected in A375 cells after treatment with vehicle control and complexes for 24 h; (**b**) Quantification of the flow cytometric results in (**a**) showing the percentage of cells with increased intracellular DCF oxidation compared to control cells. Results are the mean ± S.E.M (n = 3) *p < 0.05, **p < 0.01, ***p < 0.001.

The high-value generation of reactive oxygen species (ROS) is among the most effective agents on the imbalance of cellular redox homeostasis and is followed by cell destruction. Therefore, compounds with the ability to produce ROS have a prominent role in cellular activities. In some anticancer studies, it has been recognized that inhibition properties of the compounds on cancer cells have affected the rate of ROS generated by them^34–36^. In our study, all three complexes selected to investigate intracellular ROS produced in A375 cells and data analyzed by flow cytometry after 24 h (Fig. 5a). The height of the DCF fluorescence peaks proves that C1, C2, and C3 enhance ROS level so that it can be 189.9%, 23.5%, and 229.4% more effective than vehicle control-treated (Fig. 5b). As can be seen, this value is very low for C2 compared to the other two complexes. Hence apoptosis is proposed as the main mechanism for the death of A375 cells treated to C1 and C3^37^ and for its approval further evaluations are carried out on these two complexes. Also, the production of ROS was measured in HFF cells treated by C3 and it was observed that its value was much less than what was produced in cancer cells of A375 (Figure S13).

### Cell cycle analysis

**Figure 6:**
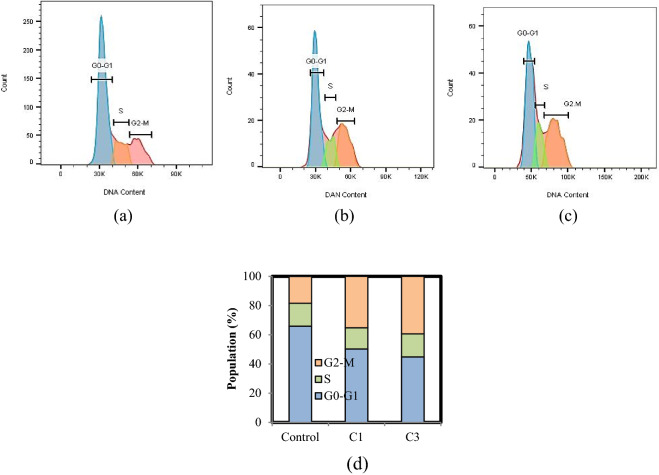
A375 cells were treated with (**a**) Vehicle control, (**b**) C1, and (**c**) C3 at IC_50_ concentrations for 24 h, after which they were stained with PI and analyzed by flow cytometry. (**d**) Populations for cell cycle distribution.

Cell cycle analysis was performed by flow cytometry to identify the percentage of time that cells spend in different phases of the cell cycle^38^. In this study, the effect of C1 and C3 on cell cycle distribution in A375 cells was evaluated to distinguish whether these complexes can exert their cytotoxic activity through alteration in cell cycle progression and/or induction of apoptosis in mentioned cells. As results indicated, both C1 (16.91%) and C3 (21.10%) were able to induce a significant rise in the cellular population of the G2-M phase in A375 cells (Fig. 6a–d) with respect to untreated control cells. Conclusively, these data suggest that alteration of the G2-M phase by complexes at IC_50_ concentrations is a potential anticancer efficacy and subsequent apoptosis induction is expected on mentioned cancer cells. For further evaluation, the effect of C3 on cell cycle distribution in HFF cells was studied and the results exhibited that it does not have a remarkable effect on the cellular population in none of the phases compared to control cells (Figure S14).

### Apoptosis evaluation by Annexin-V/PI staining

**Figure 7:**
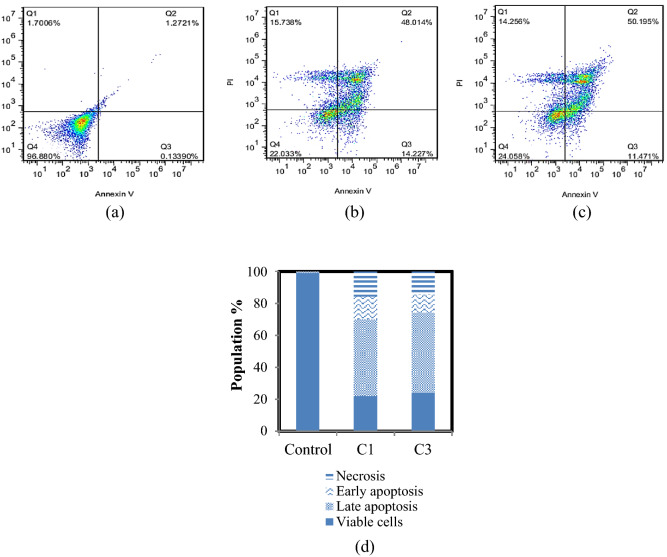
Effect of complexes on cell apoptosis for 24 h. Quantification of Annexin V and PI double-stained A375 cells after treatment with (**a**) Vehicle control, (**b**) C1, and (**c**) C3 at IC_50_ concentrations by flow cytometric. (**d**) Populations for cell apoptosis.

The type of cell death induced by the complexes was determined by flow cytometry using Annexin V-PI staining. The apoptosis assay is used to detect the ability of chemotherapeutic compounds as an anticancer drug to inhibit tumor growth. Many studies have indicated that metal complexes induced cell death mainly through apoptosis^38^. As shown in Fig. 7a–c, the lower left quadrant displays the percentage of live cells (annexin V-/PI-), the lower right shows the percentage of early apoptotic cells (annexin V + /PI-), the upper right demonstrates late apoptotic cells (annexin V + /PI +), the upper left shows necrotic cells (annexin V-/PI +). The treatment of cells with C1 and C3 at IC_50_ concentrations for 24 h, induced 14.22 and 11.47% early apoptosis and, 48.01 and 50.19% late apoptosis while these values were 0.13 and 1.27% in the control group, respectively. Quantification of the results shows that the C1 and C3 are 62.24 and 61.66% more effective than vehicle control-treated (Fig. 7d).

### Determination of MMP

**Figure 8:**
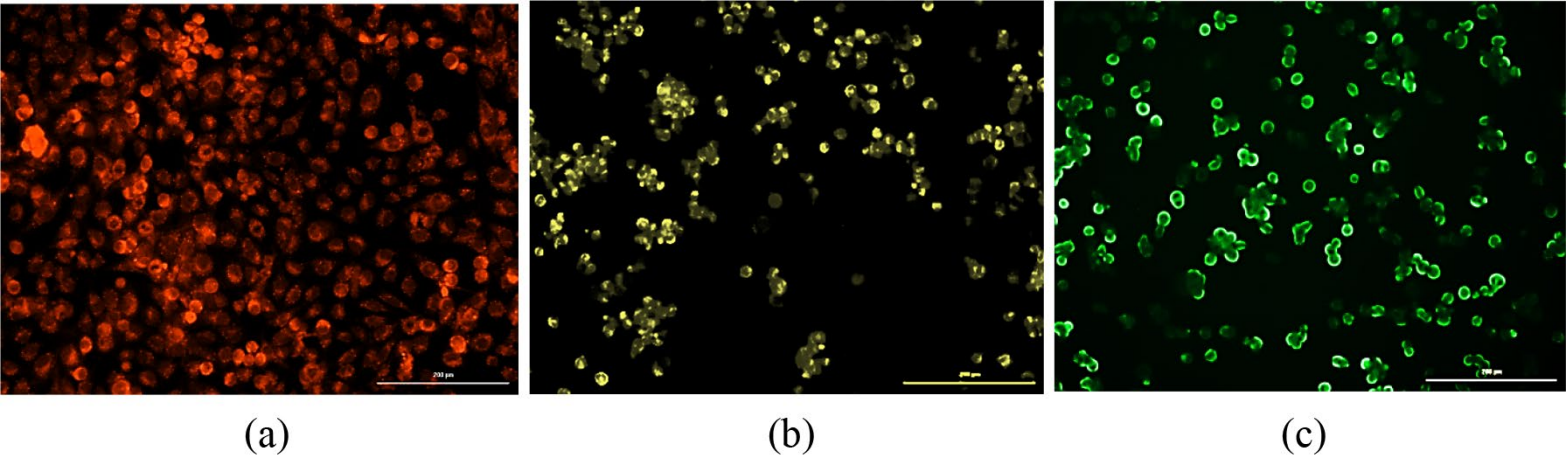
Assay of A375 cells mitochondrial membrane potential with JC-1 as fluorescence probe staining method for 24 h. (**a**) Control, (**b**) C1, and (**c**) C3.

Investigations have indicated that the formation of the tumor has been usually detected following mutations in mitochondrial genes and the generation of intracellular reactive oxygen species (ROS)^39^. Therefore it was evaluated whether the complex can induce the reduction of mitochondrial membrane potential following ROS production^40,41^. In this study, JC-1 fluorescent dye was applied to realize the cytotoxicity and signaling mechanisms of the complexes. The red fluorescence is related to the existence of JC-1 as polymer and the high mitochondrial membrane potential (Fig. 8a); while green fluorescence indicates that JC-1 presences as a monomer and it means that mitochondrial membrane potential collapses (Figs. 8b,c)^38,39^. The studies have proven that an irreversible apoptosis process can occur when the reduction of MMP and release of pro-apoptotic factors from it into the cytoplasm^42,43^. In this assessment, insignificant changes are seen in MMP of HFF cells treatment to C3 (Figure S15).

### Detection of apoptosis proteins by Western blotting

**Figure 9:**
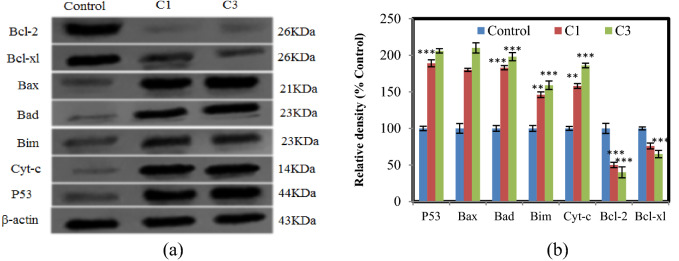
Effect of complexes on the apoptosis protein levels in A375 cells for 24 h. (**a**) Western blot analysis of p53, Bcl − 2, Bcl − xl, Bax, Bad, Bim, and Cyt-c levels after complex treatment. β − actin was used as an internal control. The blots were cropped before hybridization with antibodies during blotting. (**b**) Densitometric analysis of the expression of p53, Bcl − 2, Bcl − xl, Bax, Bad, Bim, and Cyt-c. The percentage values are those relative to the control. Results are the mean ± S.E.M (n = 3) *p < 0.05, **p < 0.01, ***p < 0.001.

To reveal the apoptotic pathway activated by the complexes, the expression levels of several anti-apoptotic and pro-apoptotic proteins were analyzed by Western blot in A375 cells. The aforementioned proteins play effective roles in inhibiting/inducing cell growth and apoptosis. In this study, both C1 and C3 significantly enhanced the level of p53. Additionally, the expression of p53 activates the regulation of Bcl-family proteins^44^. It has been realized that Bcl-2 family proteins have a prominent role in the intrinsic mitochondrial signal pathway and are strongly related to apoptotic cell death. As can be seen in Fig. 9a and S16, after incubation with the complex, the expression levels of proteins Bcl-2 and Bcl-xl were remarkably decreased while the expression of pro-apoptotic proteins Bax, Bad, Bim, and Cyt-c was gradually increased. The ratio of Bax/Bcl-2 increased from 3.60-fold to 5.25, after treatment with C1 and C3, respectively (Fig. 9b). Such an increase can lead to an enhanced permeability of the mitochondrial membrane followed by an irreversible release of apoptogenic factors.

**Figure 10:**
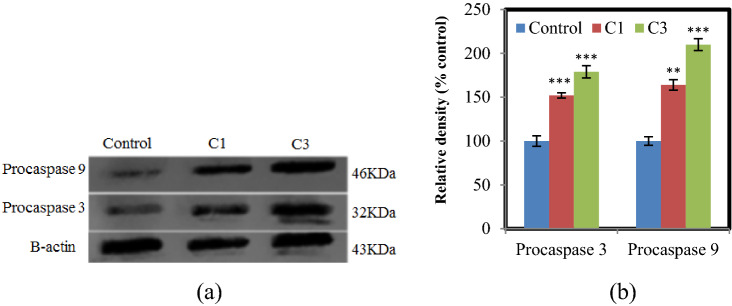
Effect of complexes on the apoptosis protein levels in A375 cells for 24 h. (**a**) Western blot analysis of procaspases 3 and 9 after complex treatment. β − actin was used as an internal control. The blots were cropped before hybridization with antibodies during blotting. (**b**) Densitometric analysis of the expression of procaspase 3 and 9. The percentage values are those relative to the control. Results are the mean ± S.E.M (n = 3) *p < 0.05, **p < 0.01, ***p < 0.001.

The caspase family is also related to cell apoptosis via the mitochondrial pathway. The studies have indicated that the generation of ROS, as well as the collapse of the mitochondrial membrane, and subsequent release of cytochrome c can lead to an increase of caspase activation^45^. So, in the present study, to perform further evaluations on the apoptotic pathways of cell death, the effects of the synthetic complexes were investigated on the expression of procaspases 9, and 3. The results confirmed that the level of procaspases 9 and 3 generally increased in the complexes-treated A375 cells (Figs. 10a,b, S17). According to the Western blot results, C1 and C3 enhanced procaspase-3 level to 52.02% and 79.97%, and procaspase-9 level to 64.12% and 110.97% compared to control, respectively^46^.

## Discussion

For the first time, thallium complexes were synthesized using protoporphyrins by Fischer and Pu¨tzer in 1929. In the next studies, the ability of thallium(III) salts to form complexes from neutral ligands was evaluated^47^, and X-ray crystal structures of some bis-thallium(I) porphyrin complexes were presented^48^. According to the reports, it was realized that thallium can form various organic and inorganic compounds with both oxidation states (I) and (III)^6^, and oxidation state (II) was observed in organometallic complexes of thallium though this state was the least seen^49^.

In our study, three new Tl(III) complexes with pyridine dicarboxylic acid derivatives were reported. Many studies have proved that the pyridine dicarboxylates family as a ligand can be suitable options to design diversity of metal complexes^28–30^. Here, electrochemical studies confirmed that these ligands, despite having no electrochemical activity, can affect the electrochemical activity of the complexes. So, the presence of only OH group in the 4-position of the pyridine ring of C2 caused that, despite having the same metal center, geometry, and counter ions, C1 and C2 to be completely different in their formal potentials. Also, the electrochemical behavior of C3 was different from C1. Such difference may be related to various geometries in these two complexes. Therefore, coordination geometry can also be an important factor in the redox activity of the complexes so that the formal potential for C3 is exhibited two and five times higher than C1 and C2, respectively. In MTT assay, it was exhibited that C1 has more potent cytotoxicity than C2 toward A375 cells. According to crystallographic data, the only difference between structures C1 and C2 was related to the presence of OH in the 4-position of the pyridine ring of C2. It seems that this group led to decreases in cytotoxicity C2 compared to C1. Therefore, it can be concluded that the type of ligand coordinated and substitutions on it has a high impact on the anti-tumor activities of inorganic compounds. Also, the stronger anti-proliferative effect of C3 compared to C1 can be related to the difference in coordination geometry and extranuclear cation in these complexes (with the same ligand coordinated).

**Figure 11:**
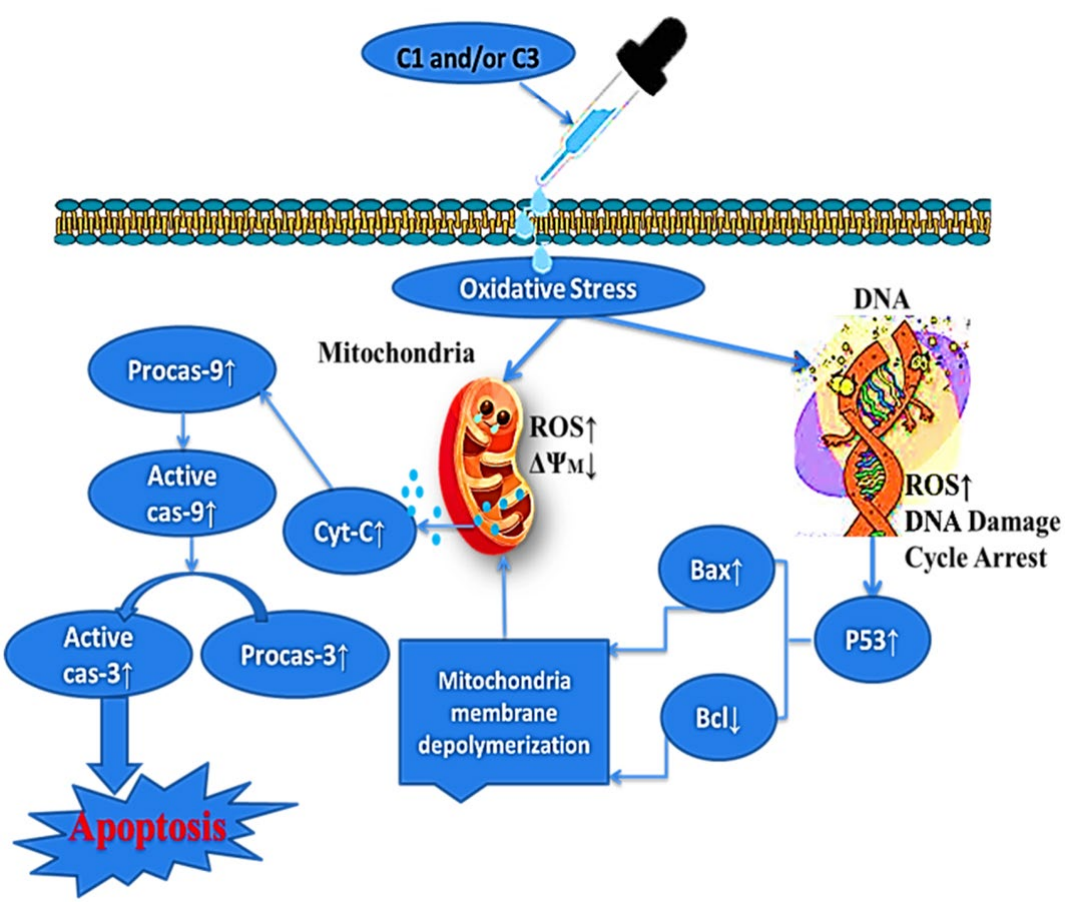
Proposed mechanism of action for the anticancer activity of C1 and/or C3 against A375 human melanoma cancer cells. The oxidative stress caused by these complexes results in the high-value production of ROS. The ROS overproduction induces the activation of the intrinsic apoptotic pathway due to changes in the mitochondrial potential. The mitochondrial damage results in cytochrome C release in the cytosol and subsequently level of procaspases 9 and 3 increases. Moreover, apoptosis via the intrinsic pathway can be induced by the tumor suppressor p53 that activates Bax in response to DNA damage.

On the other hand, the correlation of the anticancer effect of chemical compounds with their intrinsic electrochemical properties has been investigated in some studies, and it was concluded that redox-active compounds possessing higher formal potential usually represent a stronger anti-proliferative effect on cancer cells^34,50^. The results obtained from our electrochemical evaluations following previous studies in this field show that C1 and C3 with further formal potential E^0ˊ^(0.109 V) and (0.244 V) respectively exhibit higher inhibitory effects compared to C2 (–0.051) on the cancer cell lines. Such a feature can arise from the effectiveness of these compounds on the cellular redox homeostasis of cancer cells through the production of excessive values of ROS^51^. Many studies have proven that some anti-cancer compounds can interfere with the metabolic balance of ROS and enhance its level. This increase can lead to the collapse of mitochondrial membrane potential, an increase of the mitochondrial outer membrane permeability, and finally apoptosis^38^. To better understand why C1 and C3 are more cytotoxic than C2 against A375 cells, before ROS assay, cellular uptake of Tl in these cells was evaluated. The results indicated that all three complexes can penetrate easily into the A375 cells. Therefore, intracellular ROS amount was measured and the results were exhibited that ROS value generated by C2 is very lower than the other two complexes. The presence of OH group in C2 was considered as an important agent on the decrease of electrochemical activity as well as ROS production by this complex. Although the toxicity mechanism of Tl(III) is still unknown, the investigations have somewhat confirmed that its cytotoxicity may be due to creating oxidative stress states. Oxidative stress can destruct proteins, lipids, carbohydrates, and nucleotides and also, induce mutations to the DNA. Oxidative DNA damage has a remarkable role in the pathophysiology of numerous diseases, including cancer, atherosclerosis, and neurodegenerative disorder^9^. The study of operating mechanisms of thallium in various cell lines, tissues, as well as distinct animal species has proven that in most cases, the formation of ROS was the main factor for tissue damage and organ dysfunction^52^. In this study, the data obtained from cell cycle analysis and apoptosis assay confirmed that C1 and C3 could arrest cells in the G2-M phase and induce apoptotic cell death. The fluorescence microscope images indicated that these two complexes can decrease the mitochondrial membrane potential in A375 cells. Mitochondria have an important role in most eukaryotic cells to produce energy and regulate apoptosis. It was concluded that in most cases, the generation of intracellular ROS can lead to cellular damage especially the collapse of mitochondria membrane potential. The produced ROS impair the electron transport chain and lead to apoptosis^39^. Following results reported in this field, the decrease of MMP leads to the release of pro-apoptotic proteins which start the death-signaling cascade. Cytochrome c is a small hemeprotein found loosely associated with the inner membrane of the mitochondrion that can activate the caspase cascade and lead to apoptotic cell death. In our study, the expression of procaspases 9 and 3 increased in the complexes-treated A375 cells following the release of hemeprotein mentioned. Also, mitochondrial outer membrane permeabilization was proved through a mechanism involving Bcl-2 family proteins which comprises anti-apoptotic Bcl-2, and Bcl-xl, and pro-apoptotic Bax, Bim, and Bad^53,54^. In this line, p53 is a key protein in controlling cell responses to DNA damage via cell cycle arrest and apoptosis, which increases the level of responses to oxidative stress^44^. Here, p53 activation, Bax up-regulation, and Bcl-2 down-regulation, procaspase-9, and 3 expression proved that anticancer activity of C1 and C3 is through inducing mitochondria-mediated apoptosis (Fig. 11).

Destruction of cancer cells without hurting healthy cells is the vital objective of cancer therapy^55^. In MTT test, it was realized that C3 has a potent antiproliferative effect on A375 cells while its cytotoxicity was very weak on HFF cells. Therefore we reported that C3 is a selective compound toward cancer cells and it is suitable for further evaluations in the field of anticancer properties. The comparison of the results of cellular uptake of C3 in A375 and HFF cells exhibits that the permeation of C3 in A375 cells is almost two times more than normal cells. For a better assay, we measured the ROS generation by C3 in HFF cells and compared its value with ROS produced by this complex in A375 cells. In this evaluation, although ROS generated value was less in HFF cells than A375 cells, however, some were generated. Here it seems that ROS generated is not effective in destroying normal cells as well as cancer cells. Many results have indicated that cancer cells are more sensitive to ROS-induced cell death so the cellular level of ROS has a decisive role in the death of cancer cells but its effect is weak on normal cells^36,37,41^. Our results concur with these studies. By the MMP assay, it was recognized that generated ROS does not have a destructive effect on the mitochondrial membrane potential of HFF cells while mitochondrial membrane potential change in A375 cells was observed by the red color change from control to the green color in the treated cells by C3. Further study on cell cycle analysis of HFF cells treated by C3 indicated that C3 doesn’t have an important role in blocking or arresting the cell cycle in these cells.

Targeting the mitochondria is a rational explanation for the selective effects of a drug on cancer versus normal cells. Because cancer cells have increased metabolism and mitochondrial mutations, their mitochondria may be more unstable and therefore more sensitive to pharmaceutical compounds perturbations^55^. Also, it can be considered that cancer cells have a more hyperpolarised mitochondrial membrane potential than normal cells. Therefore, compounds containing positive charges may cross membranes and accumulate in the mitochondrial matrix. In this study, C3 may be more attracted to cancer cell mitochondria than normal cells^56^.

## Conclusions

Three new Tl(III) complex containing pyridine dicarboxylic acid derivatives were synthesized and their structures were determined by X-ray crystallography. Despite structural similarities, different redox couples were exhibited in electrochemical studies for the complexes. C1 and C3 indicated stronger electrochemical activity compared to C2. Also, the potent cytotoxicity effect was showed toward cancer cell lines by these two complexes. The presence of the OH group in the 4-position of the pyridine ring of C2 was considered as an effective agent on the decrease of electrochemical and anticancer properties of it. Here a direct correlation was observed between the anti-proliferative effect and the formal potential of the compounds. Since cellular uptake assay was indicated similar permeation for all three complexes in A375 cells, it seems that active redox compounds can be more effective on ROS generation and induction of apoptosis. In further evaluations, the release of cytochrome c and the increase of procaspases 9 and 3 expression were observed in A375 cells treated by C1 and C3. Therefore apoptosis through the caspase-dependent mitochondrion pathway was proposed as the main mechanism for cell death. On the other hand, our evaluations indicated that C3 can penetrate in HFF cells and generate ROS, but ROS produced either because of the low amount or its weak effectiveness in normal cells does not hurt normal cells compared to cancer cells.

## Experimental section

### Materials and apparatus

Pyridine-2,6-dicarboxylic acid, 4-hydroxy-pyridine-2,6-dicarboxylic acid, 2- aminobenzimidazole, 4-aminopyridine, and thallium(III) nitrate trihydrate were purchased from the commercial sources and were used as received. The solvents were distilled for all synthetic works. Melting points were obtained on an Electrothermal IA-9100 apparatus. The FT-IR spectra were recorded on a Bruker Vector 22 FT-IR spectrometer using KBr pellets. Electronic spectra were recorded on Specord 210, Analytik Jena spectrophotometer in the range of 200–900 nm at room temperature. The ^1^HNMR spectra were obtained from Bruker Ultrashield 400 spectrometer. Microanalyses (C, H, N) were measured with a Perkin-Elmer 2004(II) elemental analyzer. Electrochemical experiments were performed using a µAUTOLAB modular electrochemical system (ECO Chemie, Utrecht, the Netherlands) equipped with a PGSTAT type III module driven by GPES software in conjunction with a conventional three-electrode system an Ag/AgCl/3 M KCl and platinum wire as reference and counter electrode respectively and a GC as the working electrode.

### Synthesis of (2-abH)_2_[Tl(pydc)_2_(H_2_O)_2_]_2_∙11H_2_O (C1)

An aqueous solution of pyridine-2,6-dicarboxylic acid (0.15 mmol, 0.025 g) and 2- aminobenzimidazole (0.075 mmol, 0.010 g) solved in methanol were simultaneously added to a stirring aqueous solution of Tl(NO_3_)_3_.3H_2_O (0.075 mmol, 0.033 g). The solution was stirred for 3 h at room temperature. Yellow transparent crystals were obtained by slow solvent evaporation after 14 days. Yield (78%). M.p: 340 °C. Found (calc. for C_42_H_50_N_10_O_27_Tl_2_): C 32.67 (32.81), H 3.32 (3.25), N 9.17 (9.11)%. δH (400 MHz, DMSO-d^6^, 295 K): 8.36 (s, 4H, 2-abH^+^), 7.40 (d, 4H, C_5_H_3_N), 7.19 (t, 2H, C_5_H_3_N), 3.38(s, 2H, NH_2_). Selected IR bands (KBr pellet, cm^–1^): 3454, 3344 (νNH_2_), 1658 s (νCO). UV–Vis: λ_max_ (CH_3_OH, nm), 271 (Figures S18, S21 and S24).

### Synthesis of (2-abH)[Tl(hpydc)_2_(H_2_O)_2_]∙6H_2_O (C2)

This complex was synthesized by a method identical to C**1**, but 4-hydroxy-pyridine-2,6-dicarboxylic acid (0.15 mmol, 0.027 g) was used in place of pyridine-2,6-dicarboxylic acid. Finally, the solution was stirred for 3 h at room temperature. Colorless transparent crystals were obtained by slow solvent evaporation after 30 days. Yield (84%). M.p: 356 °C. Found (calc. for C_21_H_30_N_5_O_18_Tl): C 29.79 (29.82), H 3.48 (3.55), N 8.24 (8.28)%. δH (400 MHz, DMSO-d^6^, 295 K): 8.38 (s, 4H, 2-abH^+^), 8.02 (s, 4H, C_5_H_3_N), 3.41 (s, 2H, NH_2_). Selected IR bands (KBr pellet, cm^–1^): 3458, 3338 (νNH_2_), 1656 s (νCO). UV–Vis: λ_max_ (CH_3_OH, nm), 269 (Figures S19, S22 and S24).

### Synthesis of (4-apyH)[Tl(pydc)(pydcH)_2_]0.5H_2_O (C3)

This complex was prepared by a method identical to C1, but 4-aminopyridine (0.05 mmol, 0.005 g) in water was used in place 2-aminobenzimidazole. Then, the unclear solution was refluxed for 5 h at 100 °C. The white precipitate was filtered off and recrystallized from methanol. White crystals were collected after 36 days. Yield (69%). M.p: 248 °C. Found (calc. for C_26_H_28_N_5_O_17_Tl): C 35.24 (35.17), H 3.09 (3.15), N 7.76 (7.89)%. δH (400 MHz, DMSO-d^6^, 295 K): 8.36 (s, 4H, 4-apyH ^+^), 7.96 (t, 3H, C_5_H_3_N), 7.16 (d, 6H, C_5_H_3_N), 3.51 (s, 2H, NH_2_). Selected IR bands (KBr pellet, cm^–1^): 3485, 3367 (νNH_2_), 1664 s (νCO). UV–Vis: λ_max_ (CH_3_OH, nm), 297 (Figures S20, S23 and S24).

### X-ray crystallography

The X-ray measurement of single crystals of three compounds was carried out using a Bruker SMART APEX II diffractometer equipped with a CCD area detector at 298 K, with graphite-monochromated Mo-Kα radiation, λ = 0.71073 Ǻ. All refinements were done by the full-matrix least-squares method on F^2^ using the SHELX-97 program and absorption corrections were performed using the SADABS program^57–61^.

### Cell culture and cytotoxicity assay

In this study, A375 (a human melanoma), HT29 (a human colon adenocarcinoma), and HFF (a human foreskin fibroblast) cell lines were provided from the Pasteur Institute (Iran). The cells were cultured as recommended at 37 °C in a humidified atmosphere of 5% CO_2_ and 95% air in a Thermo Fisher Incubator. 10 mM stock solutions of compounds were prepared in DMSO and diluted in a fresh medium for use. The final concentration of DMSO never exceeded 0.1% (v/v). Cells were seeded into a 96-well plate and incubated for 24 h at 37 °C, 5% CO_2_, and 95% air. Then the medium was replaced with the respective medium containing ligands or Tl(III) complexes at different concentrations and incubated for 48 h. 10 μL of MTT was added and the medium was removed after 4 h of incubation. Finally, the violet crystal was solubilized in 100 μL DMSO and the absorbance was measured at 570 nm. IC_50_ value was measured by the standard method. The evaluations were carried out in triplicate^21,23^.

### Uptake of Tl(III) complexes in A375 and HFF cells

A375 cells (1 × 10^7^ cells) were treated with 10 μM C1–C3 and Tl(NO_3_)_3_.3H_2_O, respectively, for 24 h at 37 °C, 5% CO_2_, and 95% air. Cold PBS (phosphate buffer, pH = 7.4) was used for rinsing the cells three times after being collected. Cells were lysed in 1 M NaOH (1 mL) and diluted with 2% (v/v) HNO_3_ (5 mL) to determine whole-cell thallium content. The amount of thallium taken up by the cells was measured by ICP-MS. The instrument calibrates thallium using a standard solution containing 10, 50, 100, 500, and 1000 ppb thallium. This test was performed as explained on HFF cells treatment to C3^38^.

### The measurement of intracellular ROS

The generation of intracellular ROS was evaluated by reactive oxygen species assay kit. 2 mL of A375 cells (1 × 10^6^ cells/mL) were induced with C1–C3 (at IC_50_ concentrations) at 37 °C for 24 h, serum-free medium washed twice. Then, the cells were incubated in 2 mL of serum-free medium containing H2DCF-DA (2 μM) for 30 min at 37 °C, serum-free medium washed twice. Cells were collected and assayed by flow cytometer with excitation wavelength at 489 nm and emission wavelength at 524 nm, and ten thousand events were collected. The DCF fluorescence peak for this cell sample was evaluated and data were analyzed by FlowJo software. This test was performed as explained on HFF cells treatment to C3 (at the highest concentration)^38^.

### Cell cycle analysis

A375 cells were seeded in 6-well plates with 1.5 × 10^6^ cells /well and treated with the C1 and C3 at IC_50_ concentrations for 24 h. Cells were trypsinized, collected, and fixed in ice-cold 75% ethanol at − 20 °C overnight. After centrifugation, the fixed cells were rinsed with ice-cold phosphate-buffered saline (PBS) and resuspended in 0.5 mL of PBS containing 100 μg/mL DNase-free RNase A (Thermo Fisher Scientific Biosciences GmbH, St. Leon-Rot, Germany), 50 μg/mL propidium iodide (PI; Sigma Aldrich) in the dark for 30 min. The distribution of the cells in different phases of the cell cycle was determined using flow cytometry (Attune Nxt, Thermo Fisher Scientific-US) and FlowJo software v10.6.1 (https://www.flowjo.com/solutions/flowjo/downloads) was used to analyze the data. This test was performed as explained on HFF cells treatment to C3 (at the highest concentration)^62^.

#### Identification of apoptosis using Annexin-V/PI staining

Annexin V-FITC and PI staining (Annexin V/PI) apoptosis detection kit were used for assessing the percentage of dead cells by apoptosis pathway. Briefly, A375 cells were treated with C1 and C3 at IC_50_ concentrations. After 24 h of incubation, cells were washed with PBS, trypsinized, centrifuged, and re-suspended in 100 μL binding buffer at a concentration of 1 × 10^6^ cells/mL, then added 5 μL of annexin V-FITC conjugate (30 min), and 5 μL of Propidium Iodide (PI) to each cell suspension (20 min) in the dark. The samples were analyzed using a flow cytometer (Attune Nxt, Thermo Fisher Scientific-US), and the results were assessed using the FlowJo software^62^.

#### Mitochondrial membrane potential assay

Mitochondrial membrane potential analysis was determined by fluorescent dye JC-1 staining according to the manufacturer's protocol for the JC-1 assay kit. In brief, 2 mL of A375 cells (1 × 10^5^ cells/mL) were treated with IC_50_ concentrations of C1 and C3 in 6-well plates for 12 h. Cells were harvested and washed three times with PBS after 24 h of incubation. Then cells were stained with 1 mL of JC-1 stock solution (10 mg/mL). Assays were initiated by incubating A375 cells with JC-1 for 30 min at 37 °C in the dark and detected with a fluorescence microscope. This test was performed as explained on HFF cells treatment to C3 (at the highest concentration).

#### Western blotting analysis

After incubation with C1 and C3 at IC_50_ concentrations for 24 h, A375 cells were harvested and lysed in 150 μL of lysis buffer (149 μL RIPA and 1 μL PMSF), and then placed on ice for 15 min. Total proteins were extracted by centrifuging the cell sample at 10,000 rpm and 4 °C for 10 min. Then about 50 μg of purified proteins were mixed with an equal volume of electrophoresis sample buffer, and the mixture was heated for 10 min. Meanwhile, the equal protein content from the control cells was prepared in the same way. The cell sample (10 μL) was loaded onto 10% SDS-PAGE gels and then transferred onto a microporous polyvinylidene difluoride (PVDF) membrane. The membrane was blocked with 5% BSA in TBST buffer for more than 2 h. After the removal of TBST buffer, membranes were incubated with an appropriate dilution of specific primary antibodies in TBST overnight at 4 °C and then washed with TBST three times. The membranes were incubated with secondary antibodies for 1 h and washed with TBST. The immunoreactive signals were detected using an enhanced chemo-luminance kit (Pierce ECL Western Blotting Substrate) following the procedures given in the user manual^46,63^.

## Supplementary Information

Supplementary Information.
