# Supplementary material for: Novel Tl(III) complexes containing pyridine-2,6-dicarboxylate derivatives with selective anticancer activity through inducing mitochondria-mediated apoptosis in A375 cells
Source: Sci Rep. 2021 Aug 3;11:15699. doi: 10.1038/s41598-021-95278-y (PMC8333620; doi:10.1038/s41598-021-95278-y)
Supplement: Supplementary file 1 — Supplementary Information. [file 41598_2021_95278_MOESM1_ESM.docx]

**Novel Tl(III) complexes containing pyridine-2,6-dicarboxylate derivatives with selective anticancer activity through inducing mitochondria-mediated apoptosis in A375 cells**

Sara Abdolmaleki ^a^, Mohammad Ghadermazi ^a*^, Alireza Aliabadi ^b^

^a^ Department of Chemistry, Faculty of Science, University of Kurdistan, Sanandaj, Iran

^b^ Pharmaceutical Sciences Research Center, Health Institute, School of Pharmacy, Kermanshah University of Medical Sciences, Kermanshah, Iran

**Scheme** **S1.** Synthesis route of compounds


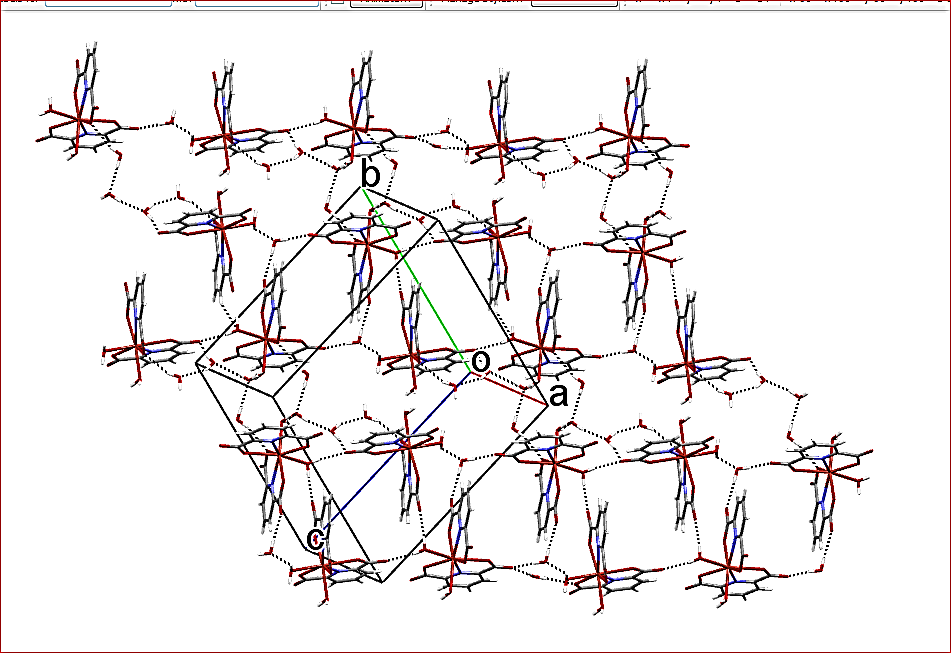


(a)


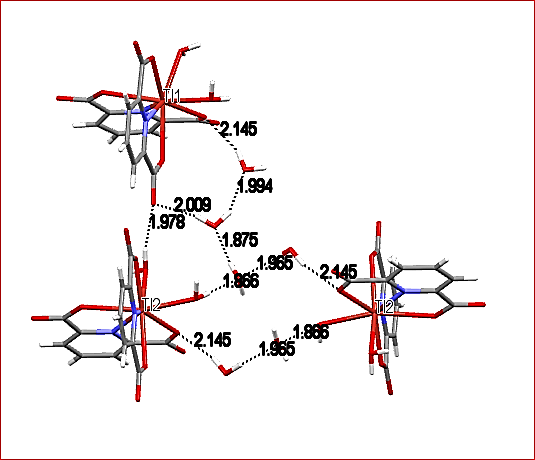

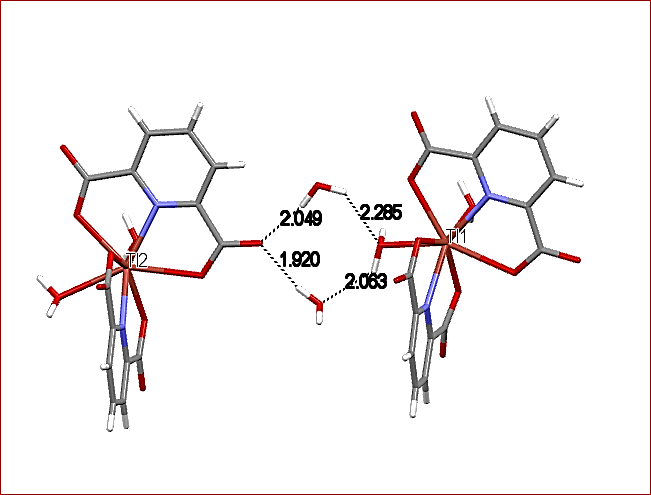


(b)

**Fig. S2.** a) Hydrogen bonds existing between layers in the unit cell; b) Formation of hydrogen bonding rings as *R*$\begin{matrix} 4 \\ 6 \end{matrix}$(16), *R*$\begin{matrix} 3 \\ 3 \end{matrix}$(10) and *R*$\begin{matrix} 3 \\ 4 \end{matrix}$(8) between carboxylate moieties and water molecules, for C1


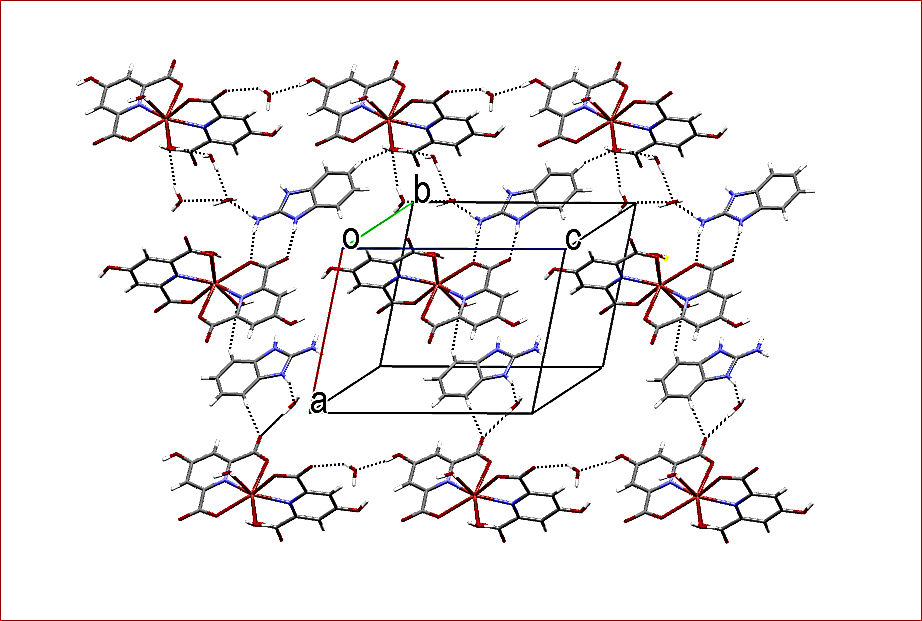


(a)


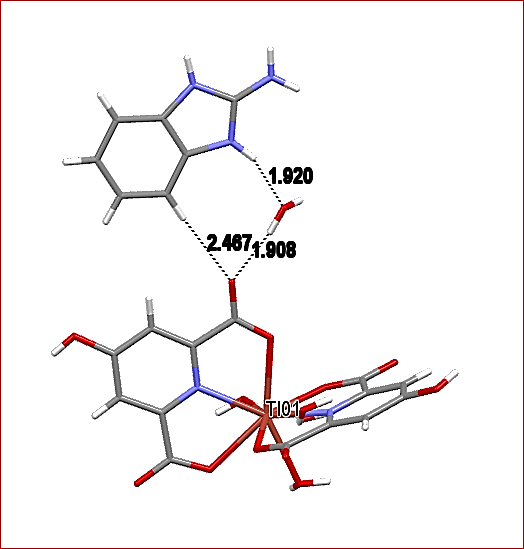

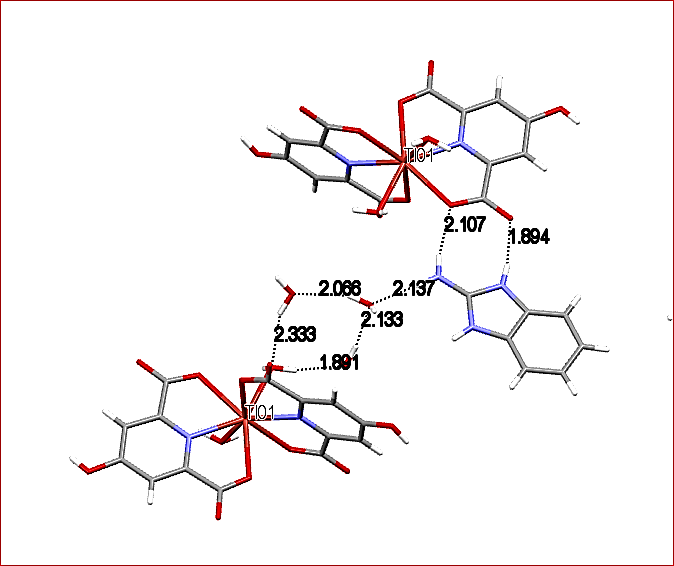


(b)

**Fig. S3.** a) Hydrogen bonds existing between layers in the unit cell; b) Formation of hydrogen bonding rings as *R*$\begin{matrix} 2 \\ 3 \end{matrix}$(8), *R*$\begin{matrix} 2 \\ 2 \end{matrix}$(8) and *R*$\begin{matrix} 4 \\ 4 \end{matrix}$(8) between carboxylate moieties, 2- aminobenzimidazole and water molecules, for C2


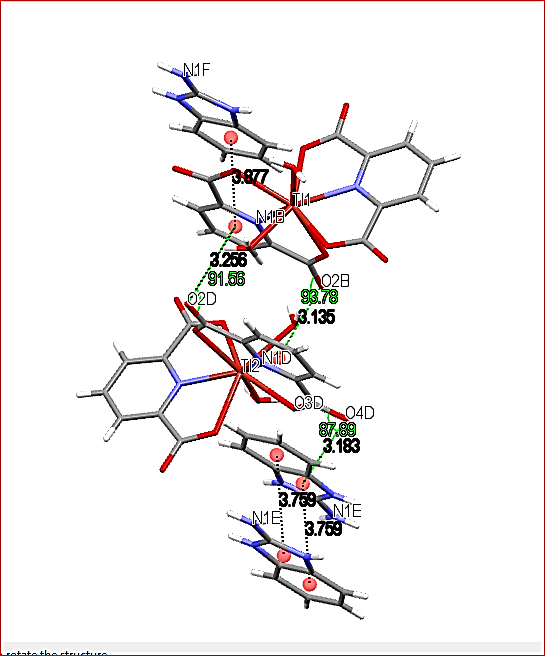


**Fig. S4.** a) C–O···π stacking between C(1D)–O(2D) and C(1B)–O(2B) with (N1B, C2B-C6B), and (N1D, C2D–C6D) rings respectively, and C(7D)–O(4D) with (2-abH)^+^, for C1


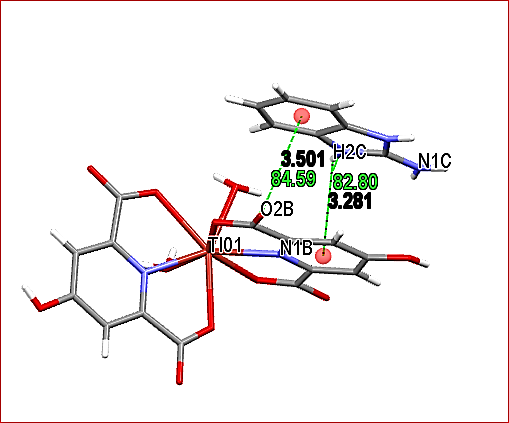


**Fig. S5.** N–H···π and C–O···π stacking interactions respectively between N(2C)–H(2C) with (N1B, C2B-C6B ) and C(1B)–O(2B) with (2-abH)^+^, for C2


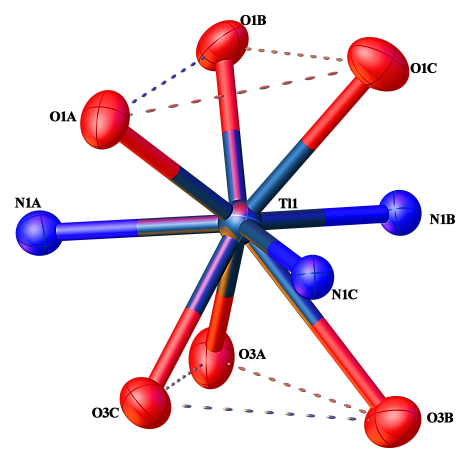


**Fig. S6.** The formation of distorted tricapped triangular prism around Tl(III), for C3


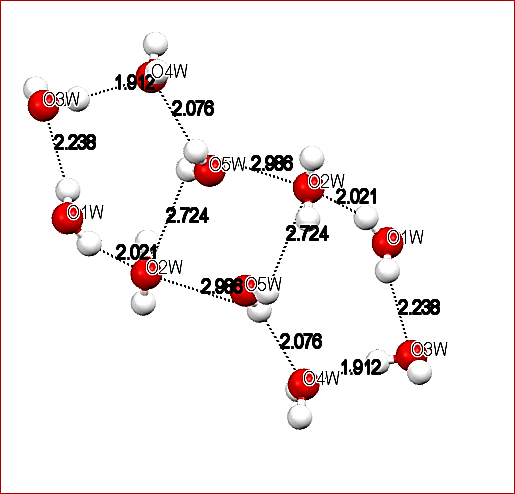


**Fig. S7.** The tetrameric and pentameric water clusters hosted, for Tl^3+^ in C3


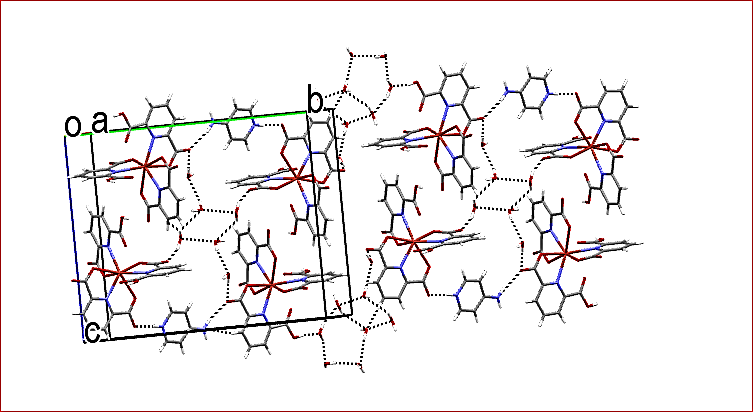


(a)


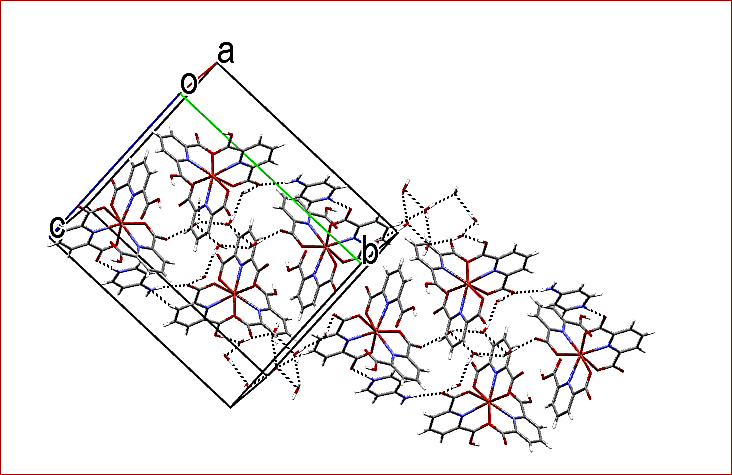


(b)

**Fig. S8.** Hydrogen bonds existing between layers in the unit cell, for C3

**1S. Stability testing of the complexes**

The stability of the complexes was evaluated by UV-Vis spectral analysis at different times in a solution containing DMSO/H_2_O at concentration 100 μM, 37 °C, and pH 7.2. The degree of decomposition over time was determined by comparing the spectra collected after 0, 24, and 48 h. The results indicated that the values of absorbance and wavelength were not affected, even after 48 h, and confirmed that the complexes are stable in the solution for at least 48 h under the test conditions.

(a) (b)

(c)

**Fig. S9.** UV spectra of complexes (100 μM) in DMSO / H_2_O after 0, 24, and 48 h a) C1, b) C2 and c) C3

**2S. Statistical analysis**

Data were reported as means±SD (number of experiments). All tests were performed in triplicates with the full agreement between the results. The statistical significance was assessed using Student's *t*-test.

(a)

(b)

(c)

**Fig. S10.** Cytotoxic effect of oxaliplatin and synthetic compounds against a) A375, b) HT29 and c) HFF cell lines. The incubated cells were treated with concentrations of 0.1–312.5 μM compounds for 48 h. Cytotoxicity was determined under the MTT method as explained. Data were registered as mean ±S.E.M (n=3)

**Fig. S11.** Cellular uptake of Tl in A375 cells. A375 cells were incubated with 10 μM of Tl(NO_3_)_3_.3H_2_O and C1–C3 for 24 h at 37 °C

**Fig. S12.** Cellular uptake of Tl in HFF cells. HFF cells were incubated with 10 μM of Tl(NO_3_)_3_.3H_2_O and C3 for 24 h at 37 °C


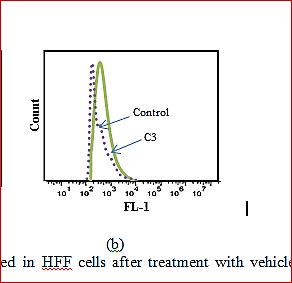


(a) (b)

**Fig. S13.** (a) Intracellular ROS was detected in HFF cells after treatment with vehicle control and C3 (at the highest concentration) for 24 h; (b) Quantification of the flow cytometric results in (a) showing the percentage of cells with increased intracellular DCF oxidation compared to control cells. Results are the mean ±S.E.M (n=3) *p < 0.05, **p < 0.01, ***p < 0.001


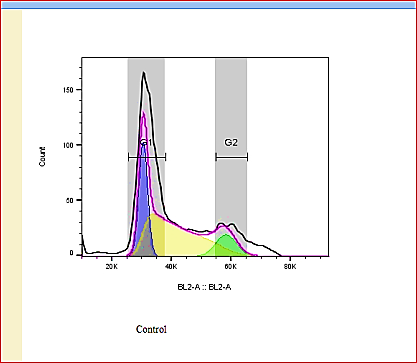

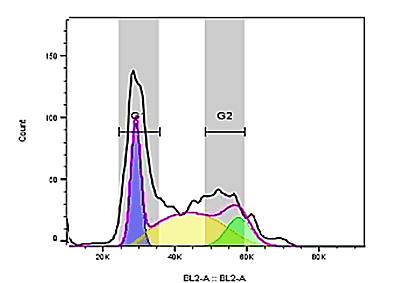


(a) (b) (c)

**Fig. S14.** HFF cells were treated with (a) Vehicle control, and (b) C3 (at the highest concentration) for 24 h, after which they were stained with PI and analyzed by flow cytometry. (c) Populations for cell cycle distribution


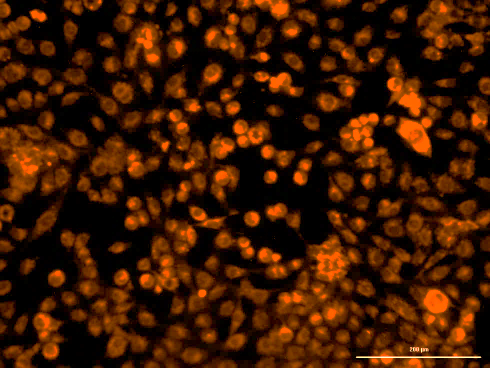

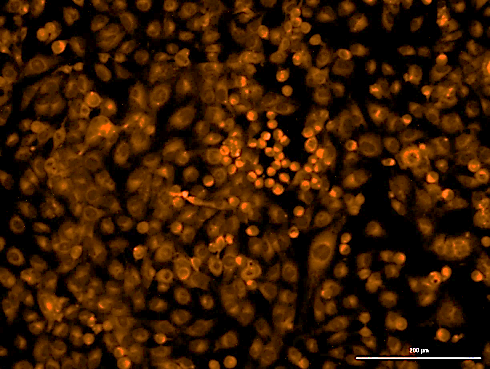


(a) (b)

**Fig. S15**. Assay of HFF cells mitochondrial membrane potential with JC-1 as fluorescence probe staining method for 24 h. (a) Control and (b) C3


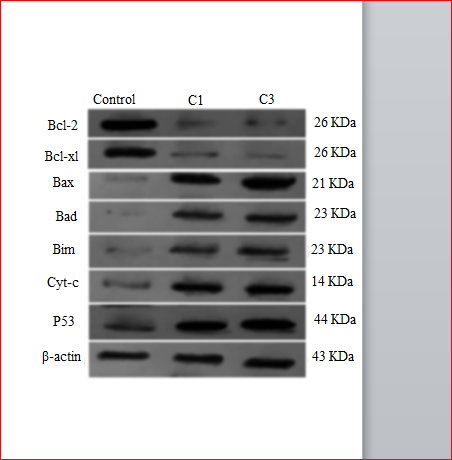

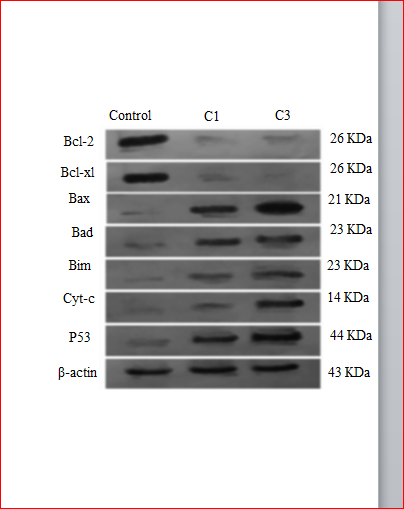


**(a) (b)**

**Fig. S16.** Effect of complexes on the apoptosis protein levels in A375 cells for 24 h. (a and b) Western blot analysis of p53, Bcl−2, Bcl−xl, Bax, Bad, Bim, and Cyt-c levels after complex treatment. β−actin was used as an internal control. The blots were cropped before hybridization with antibodies during blotting.

**
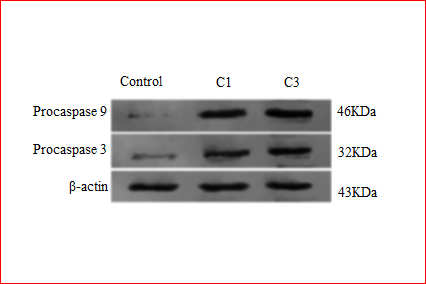
**  **
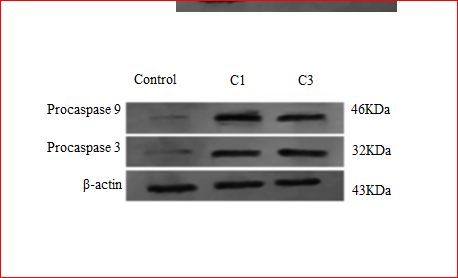
**

**(a) (b)**

**Fig. S17.** Effect of complexes on the apoptosis protein levels in A375 cells for 24 h. (a and b) Western blot analysis of procaspases 3 and 9 after complex treatment. β−actin was used as an internal control. The blots were cropped before hybridization with antibodies during blotting.

**Fig. S18**. IR spectrum of C1

**Fig. S19**. IR spectrum of C2

**Fig. S20**. IR spectrum of C3


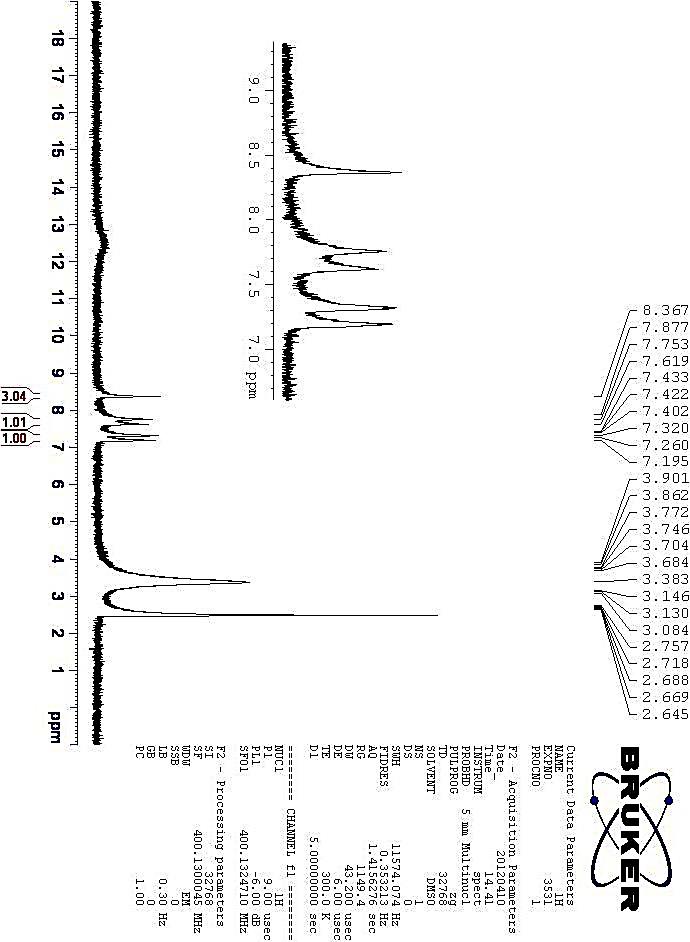


**Fig. S21**. NMR spectrum of C1


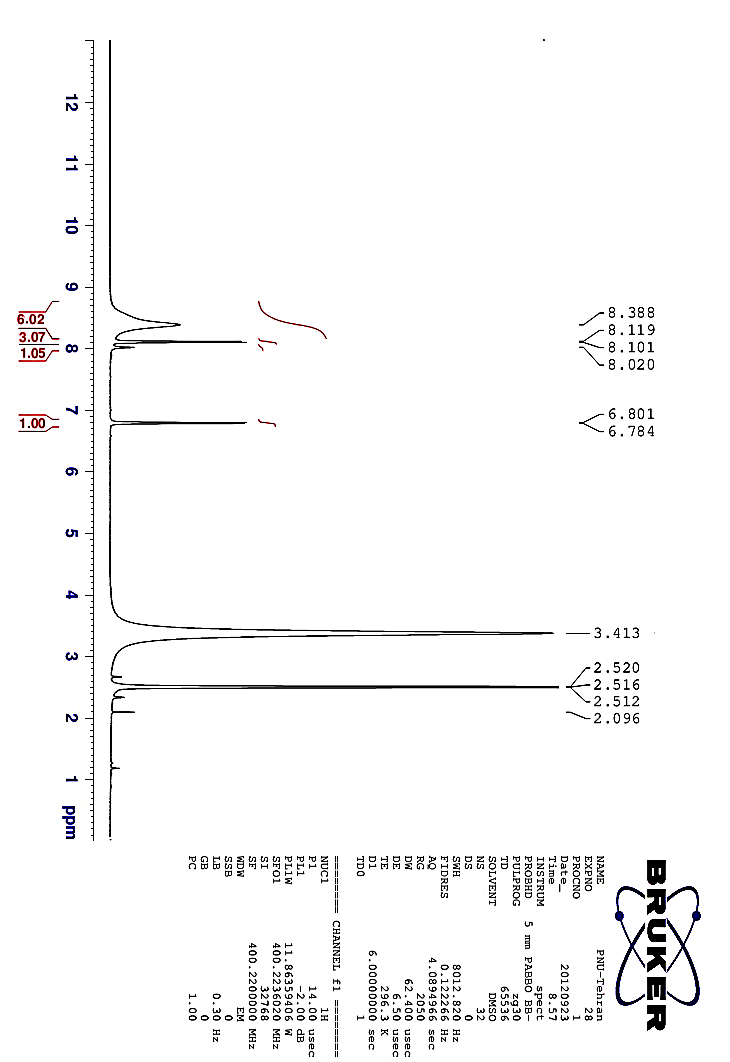


**Fig. S22**. NMR spectrum of C2


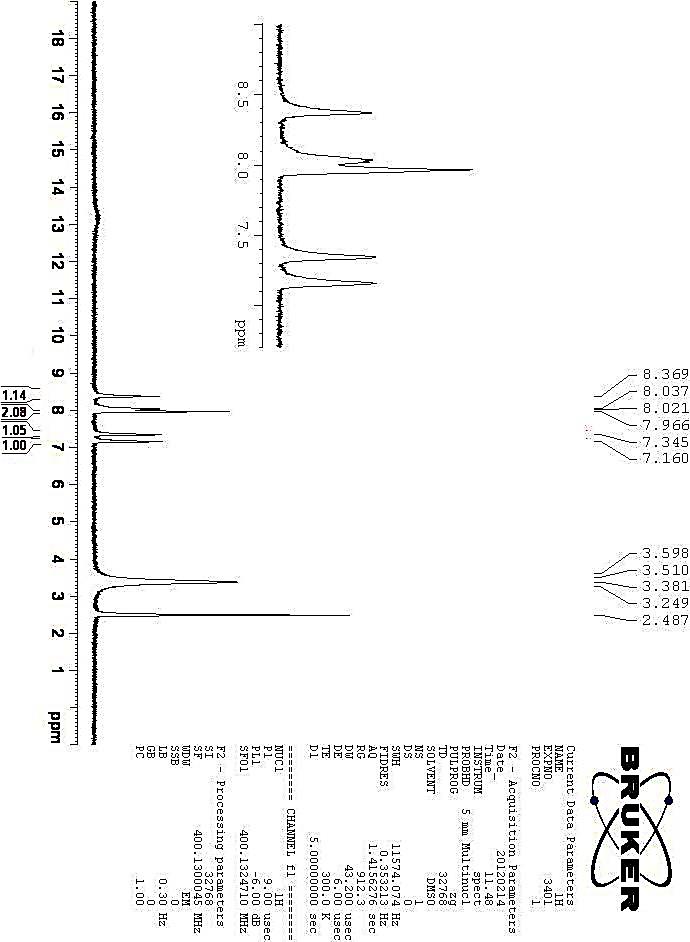


**Fig.** **S23**. NMR spectrum of C3

**Fig. S24.** UV-Vis spectra of complexes (in H_2_O)

**Table S1.** Selected bond lengths (Å) and angles (°) for the complexes

| C1 | | | | |
| --- | --- | --- | --- | --- |
|  | | | | |
| Tl1—O1A | 2.323 (3) | | **C4D—C5D** | 1.395 (5) |
| Tl1—O1B | 2.528 (2) | | **C5C—H5C** | 0.9300 |
| Tl1—O1W | 2.432 (3) | | **C5C—C6C** | 1.397 (6) |
| Tl1—O2W | 2.328 (3) | | **C5D—H5D** | 0.9300 |
| Tl1—O3A | 2.442 (3) | | **C5D—C6D** | 1.387 (5) |
| Tl1—O3B | 2.328 (3) | | **C6C—C7C** | 1.522 (6) |
| Tl1—N1A | 2.284 (3) | | **C6D—C7D** | 1.527 (5) |
| Tl1—N1B | 2.264 (3) | | **O5W—H5WA** | 0.8499 |
| Tl2—O1C | 2.300 (3) | | **O3A—C7A** | 1.267 (5) |
| Tl2—O1D | 2.535 (2) | | **O3B—C7B** | 1.255 (5) |
| Tl2—O3C | 2.457 (3) | | **O4A—C7A** | 1.240 (5) |
| Tl2—O3D | 2.267 (3) | | **O4B—C7B** | 1.232 (5) |
| Tl2—O3W | 2.490 (2) | | **N1A—C2A** | 1.328 (5) |
| Tl2—O4W | 2.374 (3) | | **N1A—C6A** | 1.336 (5) |
| Tl2—N1C | 2.268 (3) | | **N1B—C2B** | 1.350 (4) |
| Tl2—N1D | 2.269 (3) | | **N1B—C6B** | 1.339 (5) |
| O1C—C1C | 1.245 (5) | | **C1A—C2A** | 1.526 (6) |
| O1D—C1D | 1.256 (5) | | **C1B—C2B** | 1.514 (5) |
| O2C—C1C | 1.247 (5) | | **C2A—C3A** | 1.388 (6) |
| O2D—C1D | 1.247 (4) | | **C2B—C3B** | 1.368 (6) |
| O1A—Tl1—O3B | 136.86 (10) | | **C2C—N1C—Tl2** | 117.4 (2) |
| O1W—Tl1—O1B | 99.47 (10) | | **C2C—N1C—C6C** | 121.7 (3) |
| O1W—Tl1—O3A | 149.46 (11) | | **C6C—N1C—Tl2** | 120.6 (3) |
| O2W—Tl1—O1B | 148.55 (10) | | **C2D—N1D—Tl2** | 123.5 (3) |
| O2W—Tl1—O1W | 86.45 (10) | | **C6D—N1D—Tl2** | 115.9 (2) |
| O2W—Tl1—O3A | 91.52 (10) | | **C6D—N1D—C2D** | 120.6 (3) |
| O2W—Tl1—O3B | 72.20 (11) | | **O1C—C1C—O2C** | 125.3 (4) |
| O3A—Tl1—O1B | 98.02 (10) | | **O1C—C1C—C2C** | 117.5 (4) |
| O3B—Tl1—O1B | 139.25 (10) | | **O2C—C1C—C2C** | 117.2 (4) |
| O3B—Tl1—O1W | 76.02 (11) | | **O1D—C1D—C2D** | 116.8 (3) |
| O3B—Tl1—O3A | 74.37 (10) | | **O2D—C1D—O1D** | 126.3 (4) |
| N1A—Tl1—O1A | 71.08 (11) | | **O2D—C1D—C2D** | 116.9 (4) |
| N1A—Tl1—O1B | 76.43 (9) | | **N1C—C2C—C1C** | 115.4 (3) |
| N1A—Tl1—O1W | 140.16 (12) | | **N1C—C2C—C3C** | 121.2 (4) |
| O1C—Tl2—O1D | 77.27 (10) | | **N3E—C7E—C6E** | 131.0 (4) |
| O1C—Tl2—O3C | 140.19 (10) | | **C2E—C7E—N3E** | 107.6 (3) |
| O1C—Tl2—O3W | 70.53 (10) | | **C2E—C7E—C6E** | 121.4 (4) |
| O1C—Tl2—O4W | 79.68 (10) | | **H1FA—N1F—H1FB** | 120.0 |
| O3C—Tl2—O1D | 93.16 (9) | | **C1F—N1F—H1FA** | 120.0 |
| O3C—Tl2—O3W | 149.18 (10) | | **C1F—N1F—H1FB** | 120.0 |
| O3D—Tl2—O1C | 134.08 (10) | | **C1F—N2F—H2F** | 125.6 |
| C2 | | | | |
| Tl01—O1A | 2.382 (2) | **C4A—C5A** | | 1.398 (4) |
| Tl01—O1B | 2.4681 (18) | **C4B—C5B** | | 1.392 (4) |
| Tl01—O1W | 2.4234 (18) | **C5A—H5A** | | 0.9300 |
| Tl01—O2B | 2.3964 (19) | **C5A—C6A** | | 1.375 (4) |
| Tl01—O2W | 2.361 (2) | **C5B—H5B** | | 0.9300 |
| Tl01—O3A | 2.372 (2) | **C5B—C6B** | | 1.381 (3) |
| Tl01—N1A | 2.262 (2) | **C6A—C7A** | | 1.514 (4) |
| Tl01—N1B | 2.2899 (19) | **C6B—C7B** | | 1.519 (3) |
| O2B—C1B | 1.230 (3) | **O3W—H3WA** | | 0.8498 |
| O1A—C1A | 1.261 (3) | **O3W—H3WB** | | 0.8504 |
| O1B—C1B | 1.270 (3) | **O6W—H6WA** | | 0.8499 |
| O1W—H1WA | 0.8510 | **O6W—H6WB** | | 0.8497 |
| O1W—H1WB | 0.8507 | **O7W—H7WA** | | 0.8499 |
| O2A—C1A | 1.240 (3) | **O7W—H7WB** | | 0.8502 |
| O2B—C7B | 1.256 (3) | **O8W—H8WA** | | 0.8502 |
| O2W—H2WA | 0.8722 | **O8W—H8WB** | | 0.8496 |
| O3A—C7A | 1.245 (3) | **O9W—H9WB** | | 0.8506 |
| O3B—C7B | 1.247 (3) | **N1C—H1CA** | | 0.8600 |
| O4A—C7A | 1.247 (4) | **N1C—H1CB** | | 0.8600 |
| O4W—H4W | 0.8200 | **N1C—C1C** | | 1.316 (4) |
| O1A—Tl01—O1B | 78.31 (7) | **C2B—C3B—H3B** | | 120.3 |
| O1A—Tl01—O1W | 79.26 (7) | **C2B—C3B—C4B** | | 119.5 (2) |
| O1A—Tl01—O2B | 131.83 (7) | **C4B—C3B—H3B** | | 120.3 |
| O1W—Tl01—O1B | 152.77 (7) | **O4W—C4A—C3A** | | 124.4 (3) |
| O2B—Tl01—O1B | 137.78 (6) | **O4W—C4A—C5A** | | 117.2 (3) |
| O2B—Tl01—O1W | 69.42 (6) | **C3A—C4A—C5A** | | 118.4 (2) |
| O2W—Tl01—O1A | 67.45 (7) | **O5W—C4B—C3B** | | 118.2 (2) |
| O2W—Tl01—O1B | 95.94 (7) | **O5W—C4B—C5B** | | 123.2 (3) |
| O2W—Tl01—O1W | 89.52 (7) | **C3B—C4B—C5B** | | 118.7 (2) |
| O2W—Tl01—O2B | 76.41 (7) | **C4A—C5A—H5A** | | 120.4 |
| O2W—Tl01—O3A | 152.33 (7) | **C6A—C5A—C4A** | | 119.3 (3) |
| O3A—Tl01—O1A | 138.90 (7) | **C6A—C5A—H5A** | | 120.4 |
| O3A—Tl01—O1B | 98.19 (7) | **C4B—C5B—H5B** | | 120.7 |
| O3A—Tl01—O1W | 88.70 (8) | **C6B—C5B—C4B** | | 118.7 (2) |
| O3A—Tl01—O2B | 77.13 (7) | **C6B—C5B—H5B** | | 120.7 |
| N1A—Tl01—O1A | 69.63 (7) | **N1A—C6A—C5A** | | 121.2 (2) |
| N1A—Tl01—O1B | 75.71 (7) | **N1A—C6A—C7A** | | 116.0 (2) |
| N1A—Tl01—O1W | 82.15 (7) | **C5A—C6A—C7A** | | 122.8 (3) |
| N1A—Tl01—O2B | 136.55 (7) | **N1B—C6B—C5B** | | 122.1 (2) |
| O1A—Tl01—O1B | 78.31 (7) | **C2B—C3B—H3B** | | 120.3 |
| O1A—Tl01—O1W | 79.26 (7) | **C2B—C3B—C4B** | | 119.5 (2) |
| C3 | | | | |
| Tl1—O1A | 2.3243 (19) | **N1B—C2B** | | 1.342 (4) |
| Tl1—O3A | 2.746 (2) | **N1B—C6B** | | 1.342 (4) |
| Tl1—N1A | 2.391 (2) | **C2B—C3B** | | 1.379 (4) |
| Tl1—O1B | 2.2861 (18) | **C3B—C4B** | | 1.377 (4) |
| Tl1—O3B | 2.7662 (18) | **C4B—C5B** | | 1.368 (4) |
| Tl1—N1B | 2.335 (2) | **C5B—C6B** | | 1.372 (4) |
| Tl1—O1C | 2.518 (2) | **C6B—C7B** | | 1.504 (4) |
| Tl1—O3C | 2.3508 (19) | **O1C—C1C** | | 1.245 (4) |
| Tl1—N1C | 2.305 (2) | **O2C—C1C** | | 1.242 (4) |
| O1A—C1A | 1.253 (3) | **O3C—C7C** | | 1.268 (4) |
| O2A—C1A | 1.237 (3) | **O4C—C7C** | | 1.219 (3) |
| O3A—C7A | 1.201 (3) | **C1C—C2C** | | 1.514 (4) |
| O4A—C7A | 1.311 (3) | **N1C—C2C** | | 1.324 (3) |
| C1A—C2A | 1.520 (4) | **N1C—C6C** | | 1.341 (3) |
| N1A—C2A | 1.334 (3) | **C2C—C3C** | | 1.381 (4) |
| N1A—C6A | 1.335 (3) | **C3C—C4C** | | 1.380 (5) |
| C2A—C3A | 1.370 (4) | **C4C—C5C** | | 1.380 (5) |
| C3A—C4A | 1.378 (4) | **C5C—C6C** | | 1.376 (4) |
| C4A—C5A | 1.373 (4) | **C6C—C7C** | | 1.509 (4) |
| C5A—C6A | 1.364 (4) | **N1D—C1D** | | 1.334 (4) |
| O1A—Tl1—O3A | 133.28 (6) | **C5A—C6A—C7A** | | 122.6 (2) |
| O1A—Tl1—N1A | 70.06 (7) | **O3A—C7A—O4A** | | 125.0 (3) |
| O1A—Tl1—O3B | 142.74 (7) | **O3A—C7A—C6A** | | 122.1 (2) |
| O1A—Tl1—N1B | 148.33 (7) | **O4A—C7A—C6A** | | 112.8 (2) |
| O1A—Tl1—O1C | 79.48 (8) | **C1B—O1B—Tl1** | | 118.67 (18) |
| O1A—Tl1—O3C | 87.05 (8) | **C7B—O3B—Tl1** | | 112.20 (16) |
| O3A—Tl1—O3B | 66.92 (6) | **O1B—C1B—C2B** | | 117.4 (3) |
| N1A—Tl1—O3A | 63.34 (6) | **O2B—C1B—O1B** | | 126.0 (3) |
| N1A—Tl1—O3B | 120.58 (7) | **O2B—C1B—C2B** | | 116.7 (3) |
| N1A—Tl1—O1C | 138.01 (7) | **C2B—N1B—Tl1** | | 114.26 (19) |
| O1B—Tl1—O1A | 80.85 (7) | **C6B—N1B—Tl1** | | 126.95 (17) |
| O1B—Tl1—O3A | 91.28 (7) | **C6B—N1B—C2B** | | 118.6 (3) |
| O1B—Tl1—N1A | 76.47 (7) | **N1B—C2B—C1B** | | 116.8 (3) |
| O1B—Tl1—O3B | 135.06 (6) | **N1B—C2B—C3B** | | 121.4 (3) |
| O1B—Tl1—N1B | 71.70 (7) | **C3B—C2B—C1B** | | 121.8 (3) |
| O1B—Tl1—O1C | 70.60 (7) | **C4B—C3B—C2B** | | 119.6 (3) |
| O1B—Tl1—O3C | 146.84 (7) | **C5B—C4B—C3B** | | 118.7 (3) |
| O1B—Tl1—N1C | 135.90 (7) | **C4B—C5B—C6B** | | 119.4 (3) |
| N1B—Tl1—O3A | 64.25 (7) | **N1B—C6B—C5B** | | 122.1 (3) |
| N1B—Tl1—N1A | 116.65 (7) | **N1B—C6B—C7B** | | 114.6 (2) |
| N1B—Tl1—O3B | 63.49 (6) | **C5B—C6B—C7B** | | 123.3 (3) |

**Table S2.** Selected hydrogen bonds and their geometries in the crystal packing for complexes

| H···A | D–H | H···A | D···A | < D–H···A |
| --- | --- | --- | --- | --- |
| **C1** | | | | |
| **N1—H1···O6W** | 0.86 | 2.50 | 3.093(6) | 127 |
| **N1—H1···O14** | 0.86 | 2.55 | 3.192(5) | 132 |
| **O1W—H1WA···O7** | 0.86 | 1.99 | 2.812(4) | 160 |
| **N2—H2···O11i** | 0.86 | 1.87 | 2.728(4) | 177 |
| **O2W—H2WA···O5W** | 0.85 | 1.90 | 2.740(4) | 166 |
| **N3—H3A···O10i** | 0.86 | 2.22 | 3.080(5) | 173 |
| **N3—H3B···O6W** | 0.86 | 2.57 | 2.847(6) | 100 |
| **N4—H4···O2** | 0.86 | 2.37 | 3.087(5) | 141 |
| **N5—H5···O7ii** | 0.86 | 1.92 | 2.779(4) | 173 |
| **O3W—H3WA···O11A** | 0.87 | 1.92 | 2.694(10) | 148 |
| **O3W—H3WA···O11W** | 0.87 | 1.98 | 2.682(7) | 138 |
| **O3W—H3WB···O10W** | 0.87 | 1.82 | 2.632(4) | 154 |
| **N6—H6A···O5ii** | 0.86 | 2.14 | 2.968(4) | 162 |
| **N6—H6B···O9W** | 0.86 | 2.18 | 2.987(6) | 155 |
| **O4W—H4WA···O3iii** | 0.90 | 1.93 | 2.779(4) | 157 |
| **O4W—H4WB···O13W** | 0.90 | 2.07 | 2.739(5) | 130 |
| **O7W—H7WA···O13i** | 0.85 | 2.05 | 2.888(5) | 168 |
| **O7W—H7WB···O8** | 0.85 | 2.58 | 3.285(4) | 141 |
| **O7W—H7WB···O9** | 0.85 | 2.04 | 2.859(4) | 161 |
| **O10W—H10A···O15iv** | 0.85 | 2.06 | 2.891(5) | 165 |
| **O10W—H10B···O1iii** | 0.85 | 2.22 | 3.002(4) | 153 |
| **O10W—H10B···O4Wiii** | 0.85 | 2.31 | 2.953(5) | 133 |
| **O8W—H8WA···O9W** | 0.85 | 1.99 | 2.787(7) | 155 |
| **O8W—H8WB···O5Wv** | 0.85 | 2.18 | 2.719(6) | 122 |
| **O9W—H9WA···O12A** | 0.85 | 2.10 | 2.74(2) | 132 |
| **O9W—H9WA···O12W** | 0.85 | 2.28 | 2.905(14) | 131 |
| **O13W—H13A···O11** | 0.85 | 2.01 | 2.803(4) | 154 |
| **O13W—H13B···O15vi** | 0.85 | 1.93 | 2.758(5) | 163 |
| **O9W—H9WB···O6** | 0.85 | 2.16 | 2.954(6) | 155 |
| **O6W—H6WA···O8** | 0.85 | 2.14 | 2.973(6) | 165 |
| **O6W—H6WB···O5Wvii** | 0.85 | 1.97 | 2.808(6) | 171 |
| **O5W—H5WA···O7W** | 0.85 | 1.89 | 2.737(5) | 171 |
| **O5W—H5WB···O8Wv** | 0.85 | 1.87 | 2.719(6) | 177 |
| **O1W—H1WA···N8** | 0.86 | 2.61 | 2.916(4) | 102 |
| **O3W—H3WA···O6** | 0.87 | 2.40 | 2.740(4) | 104 |
| **C26—H26···O10Wiii** | 0.93 | 2.52 | 3.276(5) | 138 |
| **C41—H41···O9ii** | 0.93 | 2.34 | 3.021(5) | 130 |
| **C42—H42···O10Wviii** | 0.93 | 2.59 | 3.492(5) | 162 |
| Symmetry codes: (i) *x−1, y, z*; (ii) *x+1, y, z*; (iii) *−x+1, −y+1, −z+1*; (iv) *x, y−1, z*; (v) *−x+1, −y+1, −z*; (vi) *−x+1, −y+2, −z+1*; (vii) *−x, −y+2, −z*; (viii) *x, y+1, z*. | | | | |
| **C2** | | | | |
| **N2F—H2F···O13W** | 0.8600 | 1.9100 | 2.734(16) | 159.00 |
| **N3F—H3F···O2D** | 0.8600 | 1.8800 | 2.73(2) | 169.00 |
| **O5A—H5A···O3Di** | 0.8200 | 1.7600 | 2.567(17) | 167.00 |
| **O5B—H5B···O1Wii** | 0.8200 | 1.8800 | 2.698(15) | 171.00 |
| **O5C—H5C···O3Biii** | 0.8200 | 1.8000 | 2.616(18) | 170.00 |
| **N1F—H1FA···O1D** | 0.8600 | 2.1100 | 2.96(2) | 168.00 |
| **N1F—H1FB···O9W** | 0.8600 | 2.1600 | 3.016(16) | 176.00 |
| **O10W—H10A···O13W** | 0.8500 | 1.9100 | 2.736(8) | 164.00 |
| **O10W—H10B···O8W** | 0.8500 | 2.0900 | 2.887(8) | 156.00 |
| **O1W—H1WA···O21W** | 0.8500 | 2.2500 | 2.913(13) | 135.00 |
| **O11W—H11A···O8W** | 0.9700 | 1.8400 | 2.752(13) | 155.00 |
| **O11W—H11B···O7W** | 0.9900 | 2.0500 | 2.907(13) | 144.00 |
| **O12W—H12A···O2Dii** | 0.9400 | 1.7300 | 2.668(16) | 172.00 |
| **O12W—H12B···O5Wiv** | 0.9400 | 1.9700 | 2.775(12) | 143.00 |
| **O13W—H13A···O4Cv** | 0.8500 | 1.9000 | 2.690(17) | 153.00 |
| **O13W—H13B···O4Di** | 0.8500 | 1.9000 | 2.714(16) | 160.00 |
| **O2W—H2WB···O21W** | 0.8500 | 2.3700 | 3.160(14) | 155.00 |
| **O3W—H3WB···O4W** | 0.8500 | 2.5000 | 3.058(7) | 124.00 |
| **O3W—H3WB···O5W** | 0.8500 | 2.3300 | 2.789(8) | 114.00 |
| **O4W—H4WA···O4Avi** | 0.8500 | 2.0300 | 2.780(13) | 147.00 |
| **O4W—H4WB···N1Evii** | 0.8500 | 2.2200 | 2.981(17) | 148.00 |
| **O5W—H5WB···O6W** | 0.8500 | 1.9700 | 2.731(8) | 149.00 |
| **O6W—H6WA···O4Biii** | 0.8500 | 1.9600 | 2.763(18) | 157.00 |
| **O21W—H21A···O2W** | 0.9400 | 2.2600 | 3.160(14) | 162.00 |
| **O21W—H21B···O1W** | 0.9400 | 2.0500 | 2.913(13) | 152.00 |
| **O6W—H6WB···N2Evii** | 0.8500 | 2.0000 | 2.764(15) | 149.00 |
| **O22W—H22A···O1C** | 0.9100 | 2.2800 | 2.646(14) | 103.00 |
| **O22W—H22A···O10Wviii** | 0.9100 | 2.0300 | 2.786(11) | 139.00 |
| **O22W—H22B···O2Bix** | 0.9100 | 2.2100 | 2.689(17) | 112.00 |
| **O7W—H7WA···O5Wiv** | 0.8500 | 1.9200 | 2.735(8) | 161.00 |
| **O8W—H8WA···O11W** | 0.8500 | 2.0900 | 2.752(13) | 134.00 |
| **O9W—H9WA···O4Cv** | 0.8500 | 2.6000 | 2.866(16) | 100.00 |
| **C4E—H4E···O3C** | 0.9300 | 2.5600 | 3.165(19) | 123.00 |
| **C3F—H3FA···O4Di** | 0.9300 | 2.4700 | 3.39(2) | 169.00 |
| Symmetry codes: (i) *x+1, y−1, z*; (ii) *x+1, y, z+1*; (iii) *x−1, y+1, z*; (iv) *x+1, y, z*; (v) *x, y−1, z*; (vi) *x, y+1, z*; (vii) *x, y, z+1*; (viii) *x−1, y, z*; (ix) *x−1, y, z−1*. | | | | |
| **C3** | | | | |
| **N1D—H1DA···O3Ci**  **N1D—H1DB···O2Aii** | 0.86  0.86 | 2.15  2.08 | 2.985(3)  2.929(4) | 164  167 |
| **O4A—H4A···O1W**  **O4B—H4B···O3W** | 0.85  0.85 | 1.71  1.77 | 2.555(3)  2.611(3) | 171  172 |
| **N4D—H4D···O2Ciii**  **O1W—H1WA···O3Wiv**  **O1W—H1WB···O2W**  **O2W—H2WA···O2Biii** | 0.86  0.85  0.85  0.85 | 1.85  2.24  2.02  2.23 | 2.704(4)  3.039(2)  2.805(3)  2.903(3) | 176  156  153  136 |
| **O2W—H2WB···O5Wiv**  **O3W—H3WA···O1Biii** | 0.85  0.85 | 2.36  2.54 | 2.904(3)  3.293(2) | 122  148 |
| **O3W—H3WA···O1Ciii**  **O3W—H3WB···O4W** | 0.85  0.85 | 2.29  1.91 | 3.002(3)  2.7594(19) | 142  174 |
| **O4W—H4WB···O1Aiii**  **O4W—H4WB···O2Aiii** | 0.85  0.85 | 2.58  1.89 | 3.154(3)  2.734(3) | 126  176 |
| **O4W—H4WA···O4C**  **O5W—H5WB···O4W**  **C3A—H3A···N1Dv**  **C4C—H4C···O5Wvi**  **C5D—H5D···O1Bvii** | 0.85  0.85  0.93  0.93  0.93 | 1.87  2.08  2.62  2.53  2.42 | 2.720(3)  2.753(2)  3.526(4)  3.367(4)  3.131(4) | 176  136  166  150  133 |
| Symmetry codes: (i) *x, −y+1/2, z−3/2*; (ii) *−x, y−1/2, −z+1/2*; (iii) *x+1, y, z*; (iv) *x, −y+1/2, z−1/2*; (v) *−x, y+1/2, −z+1/2*; (vi) *−x+1, −y+2, −z*; (vii) *x+1, −y+1/2, z−3/2*. | | | | |
|  | | | | |

**3S. Appendix A. Supplementary data**

CCDC 2052039, 2052040, and 875455 contain the supplementary crystallographic data for the **C1**, **C2,** and **C3**, respectively. These data can be obtained free of charge via http://www.ccdc.cam.ac.uk/conts/retrieving.html, or from the Cambridge Crystallographic Data Centre, 12 Union Road, Cambridge CB2 1EZ, UK; fax: (+44) 1223–336-033; or e-mail: [deposit@ccdc.cam.ac.uk](mailto:deposit@ccdc.cam.ac.uk).

l
